# Targeted Normoxemia and Supplemental Oxygen–Free Days in Critically Injured Adults: A Stepped-Wedge Cluster Randomized Clinical Trial

**DOI:** 10.1001/jamanetworkopen.2025.2093

**Published:** 2025-03-31

**Authors:** David J. Douin, John D. Rice, Erin L. Anderson, Conner L. Jackson, Alex C. Cheng, Mengli Xiao, Jessica Cwik, Laurel E. Beaty, Jessica L. Wild, Mohamud R. Daya, Pratik B. Doshi, Shannon C. Eastham, Michael D. Goodman, Scott R. Gunn, Jason S. Haukoos, Jessica A. Hudson, Jan O. Jansen, Jason T. McMullan, Julie A. Rizzo, Martin A. Schreiber, Wesley H. Self, Matthew W. Semler, Aimee Steinwand, Nicole Werner, Vikhyat S. Bebarta, Steven G. Schauer, Adit A. Ginde

**Affiliations:** 1 Department of Anesthesiology, University of Colorado School of Medicine, Aurora; 2 Department of Biostatistics, University of Michigan School of Public Health, Ann Arbor; 3 Department of Emergency Medicine, University of Colorado School of Medicine, Aurora; 4 Department of Biostatistics and Informatics, Colorado School of Public Health, University of Colorado Anschutz Medical Campus, Aurora; 5 Department of Biomedical Informatics, Vanderbilt University Medical Center, Nashville, Tennessee; 6 Department of Emergency Medicine, Oregon Health & Science University, Portland; 7 Department of Emergency Medicine, McGovern Medical School at The University of Texas Health Science Center, Houston; 8 Department of Surgery, Vanderbilt University Medical Center, Nashville, Tennessee; 9 Department of Surgery, University of Cincinnati College of Medicine, Cincinnati, Ohio; 10 Department of Critical Care Medicine, University of Pittsburgh Medical Center, Pittsburgh, Pennsylvania; 11 Department of Emergency Medicine, Denver Health Medical Center, Denver, Colorado; 12 Department of Surgery, University of Alabama at Birmingham, Birmingham; 13 Department of Emergency Medicine, University of Cincinnati College of Medicine, Cincinnati, Ohio; 14 Department of Surgery, Brooke Army Medical Center, Joint Base San Antonio-Fort Sam Houston, San Antonio, Texas; 15 Donald D. Trunkey Center for Civilian and Combat Casualty Care, Oregon Health & Science University, Portland; 16 Vanderbilt Institute for Clinical and Translational Research, Vanderbilt University Medical Center, Nashville, Tennessee; 17 Division of Allergy, Pulmonary, and Critical Care Medicine, Department of Medicine, Vanderbilt University Medical Center, Nashville, Tennessee; 18 Department of Surgery, Denver Health Medical Center, Denver, Colorado; 19 Department of Surgery, University of Wisconsin School of Medicine and Public Health, Madison; 20 Center for Combat Medicine and Battlefield Research, University of Colorado School of Medicine, Aurora; 21 US Army Institute of Surgical Research, Joint Base San Antonio-Fort Sam Houston, San Antonio, Texas; 22 Department of Emergency Medicine, Brooke Army Medical Center, Joint Base San Antonio-Fort Sam Houston, San Antonio, Texas

## Abstract

**Importance:**

Supplemental oxygen is fundamental to caring for critically injured adults but can expose them to excess inspired oxygen.

**Objective:**

To determine the safety and effectiveness of targeting normoxemia in critically ill trauma patients.

**Design, Setting, and Participants:**

This multicenter, stepped-wedge, cluster randomized clinical trial compared targeted normoxemia (defined as a peripheral oxygen saturation [Spo_2_] of 90% to 96%) with usual care among adult trauma patients admitted to an intensive care unit (ICU) at 8 level I trauma centers across the US. These trauma centers were randomized at 3-month intervals when they crossed over from usual care to targeting normoxemia. Eligible patients were enrolled between July 15, 2020, and November 14, 2022. All statistical analyses were performed from April 2023 to November 2024 according to intention-to-treat approach.

**Intervention:**

In the usual care group, supplemental oxygen was determined by treating clinicians. In the targeted normoxemia group, a multimodal educational and informatics intervention encouraged decreasing the supplemental oxygen administered whenever Spo_2_ exceeded 96%.

**Main Outcomes and Measures:**

The primary outcome was supplemental oxygen–free days (SOFDs), defined as the number of days alive and not receiving supplemental oxygen through day 28. Safety outcomes included hypoxemia (defined as Spo_2_ <88%) during the ICU admission, in-hospital mortality, and adverse events.

**Results:**

A total of 12 487 patients were enrolled (mean [SD] age, 51.7 [21.1] years; 8799 males [70.5%]; mean [SD] Injury Severity Score, 19.6 [12.0]). The proportion of ICU time spent in normoxemia increased from 56.2% in the usual care group to 71.6% in the targeted normoxemia group. Hyperoxemia (defined as Spo_2 _>96%) decreased from 42.4% in the usual care group to 26.7% in the targeted normoxemia group, and hypoxemia was similar between groups (1.1% vs 1.1%). The raw mean (SD) number of SOFDs was 19.6 (10.3) days for the targeted normoxemia group and 17.5 (10.4) days for the usual care group (adjusted mean difference [AMD], 0.32 [95% CI, −0.37 to 1.00] days; *P* = .30). Among patients not receiving mechanical ventilation at ICU admission, mean SOFDs were greater in the targeted normoxemia group than in the usual care group (22.6 [8.30] days vs 20.6 [8.86] days; AMD, 0.75; 95% CI, 0.00-1.50 days). The mean (SD) time for weaning to room air was 1.6 (3.2) days for the targeted normoxemia group and 2.7 (4.0) days for the usual care group (adjusted hazard ratio [AHR], 1.23; 95% CI, 1.13-1.33 days). In-hospital mortality to day 90 occurred in 563 patients (9.9%) in the targeted normoxemia and 732 patients (10.7%) in the usual care group (AHR, 1.05; 95% CI, 0.83-1.33). No adverse events were reported in either group.

**Conclusions and Relevance:**

This randomized clinical trial showed that targeting normoxemia did not increase the number of SOFDs but safely reduced supplemental oxygen use among critically ill trauma patients.

**Trial Registration:**

ClinicalTrials.gov Identifier: NCT04534959

## Key Points

**Question:**

Does targeting normoxemia, defined as a peripheral oxygen saturation of 90% to 96%, safely increase the number of supplemental oxygen–free days (SOFDs) among critically injured adults?

**Findings:**

In this cluster randomized clinical trial involving 12 487 critically ill trauma patients, targeting normoxemia safely reduced use of supplemental oxygen but did not increase SOFDs through day 28. Among patients not receiving mechanical ventilation, targeting normoxemia increased mean SOFDs.

**Meaning:**

The findings indicate that targeting normoxemia can safely reduce supplemental oxygen use without affecting mortality or time spent in hypoxemia and can increase the number of SOFDs among critically ill trauma patients not receiving invasive mechanical ventilation.

## Teaser

This cluster randomized clinical trial evaluates the clinical outcomes of achieving normoxemia in adult patients with traumatic injury.

## Introduction

Critically ill patients commonly receive supplemental oxygen to enhance tissue oxygen delivery and prevent hypoxemia.^1,2^ Higher oxygenation targets may provide a margin of safety against hypoxemia but increase the risk of exposure to excess fraction of inspired oxygen (FIO_2_) and hyperoxemia, which can cause oxidative damage and tissue inflammation.^3,4,5,6,7^ Excessive oxygen supplementation is common in critically ill patients,^8,9^ may be harmful,^10,11,12^ and creates logistical challenges in resource-limited settings.^13^ Conserving oxygen by safely reducing the use of supplemental oxygen can also improve logistics in austere settings where the oxygen supply is limited.

Several recent randomized clinical trials have examined oxygenation targets among critically ill adults primarily without traumatic injury.^14,15,16,17,18,19,20^A meta-analysis of these trials concluded that conservative oxygen targets were not associated with reduced mortality in patients receiving mechanical ventilation in the intensive care unit (ICU), and further trials were recommended to investigate oxygen targets in specific populations.^21^ Observational studies have suggested an association between targeted normoxemia (defined as peripheral oxygen saturation measured by pulse oximetry [Spo_2_] of 90%-96% or Pao_2_ of 60-100 mm Hg) and improved clinical outcomes among critically ill trauma patients.^22,23,24,25^ However, high-quality interventional trials (with improved estimates of treatment effect) that compare oxygenation targets in trauma patients with vs without mechanical ventilation are lacking.^12^ In a single-center pilot trial, targeting normoxemia increased the time spent in normoxemia and decreased the use of supplemental oxygen in critically ill trauma patients, but the effect of targeted normoxemia on clinical outcomes remains unknown.^26^

We conducted the Strategy to Avoid Excessive Oxygen (SAVE-O2) trial to determine the safety and effectiveness of targeting normoxemia in critically ill trauma patients. We hypothesized that targeting normoxemia would safely reduce exposure to hyperoxemia and increase the number of days alive and without supplemental oxygen.

## Methods

### Design and Oversight

SAVE-O2 was a multicenter, stepped-wedge, cluster randomized clinical trial^27^ conducted at 8 level I trauma centers geographically distributed throughout the US. The Colorado Multiple Institutional Review Board approved the trial and waived the informed consent requirement for several reasons, including that the trial intervention represented no more than minimal risk to participants and no direct contact occurred between investigators and participants or their surrogates. Required military second-level review was conducted by the Defense Health Agency Office of Human and Animal Research Oversight. The trial was overseen by an independent safety monitor. We followed the Consolidated Standards of Reporting Trials (CONSORT) reporting guideline and the CONSORT extension for the stepped-wedge cluster randomized trial.^28^

A list of the 8 participating sites, the full trial protocol, and statistical analysis plan are provided in Supplement 1.^27^ We did not modify any features of the trial (eg, no changes in site-crossover order) after it began. We sought to evaluate the superiority of targeted normoxemia compared with usual care in critically injured adults. Because the intervention required hospital-level involvement from multiple disciplines, we performed randomization at the site level. Site-level randomization also minimized the potential for contamination between participants before and after the intervention was implemented. Before the trial, none of the sites had existing protocols to promote a targeted normoxemia strategy.

### Population and Randomization

From July 15, 2020, to November 14, 2022, we enrolled adult patients who met the criteria for entry into state or national trauma registries and were admitted to a surgical or trauma ICU within 24 hours of arrival at a participating hospital. We excluded patients who were younger than 18 years, pregnant, incarcerated, or transferred from another hospital (eMethods in Supplement 2).

We randomly assigned each site to the sequence when crossover from providing usual care to targeting normoxemia occurred. Crossover occurred every 3 months at each of the 8 sites, for a total duration of 28 months. The first crossover was initiated 3 months after the start of data collection. Crossover included a 1-month run-in period, during which clinical staff engaged in educational activities and training to increase their familiarity and compliance with targeted normoxemia protocols. Patients admitted during the run-in period were not included in the final analysis.

### Intervention

The trial intervention targeted normoxemia by titrating supplemental oxygen in trauma patients in the ICU receiving mechanical ventilation vs not receiving mechanical ventilation. We defined normoxemia as an Spo_2_ of 90% to 96% or a Pao_2_ of 60 to 100 mm Hg based on a modified Delphi consensus approach that included experts at enrolling sites.^29^ In the usual care group, supplemental oxygen administration was determined by the treating clinicians. In the targeted normoxemia group, a multimodal educational and informatics intervention encouraged decreasing the amount of supplemental oxygen whenever Spo_2_ exceeded 96%. The intervention was comprehensive and initiated immediately on patient arrival to the emergency department (ED).

During usual care, oxygen titration was at the discretion of the site. Therefore, heterogeneity in usual care approach to oxygen titration existed between sites and individual clinicians.

Delivery of targeted normoxemia was previously described in detail.^27^ Briefly, the intervention included initial and ongoing clinician and staff education as well as an electronic health record (EHR) best-practice alert (eFigure 1 in Supplement 2). These alerts generally notified clinicians when a patient had sustained hyperoxemia (defined as Spo_2 _>96%) while receiving supplemental oxygen, with a nudge to decrease supplemental oxygen. The intervention protocol provided nonbinding recommendations to clinicians to decrease the FIo_2_ or supplemental oxygen flow rate for patients with hyperoxemia for 30 minutes or more to achieve the target Spo_2_ (90%-96%). The protocol recommended downtitration of oxygen by increments of 10% FIo_2_ for patients receiving mechanical ventilation and by 1 to 2 L per minute for patients not receiving mechanical ventilation until normoxemia was achieved or an FIo_2_ of 21% or room air was reached (eFigure 2 in Supplement 2).

If a treating clinician determined that another oxygenation target would be best for the patient’s care, the oxygenation target for that patient could be modified. The trial protocol directed oxygen titration only during the patient’s index ICU admission. Data collection continued after patients were transferred or discharged from the ICU, but their oxygenation titration was no longer actively managed as part of the trial protocol.

### Data Collection

We collected patient-level data on baseline characteristics and in-hospital outcomes from each site’s EHR and state trauma registries. Study data included repeated Spo_2_, Pao_2_, and FIo_2_ measurements. For patients not receiving mechanical ventilation, we converted oxygen volume to FIO_2_ by adding 3.5% for each liter per minute, starting from 21% (room air).^30^ Trial personnel blinded to group assignments collected prespecified data elements manually from the EHR using standardized operating procedures, including home supplemental oxygen use and Glasgow Outcomes Scale (GOS; score range: 1 [dead] to 5 [good recovery or neurologic outcome]) score at discharge. Full data collection protocols have been published previously.^27^

### Outcome Measures

Time 0 for all outcomes was the patient time of arrival to the ED at each site. The primary outcome was supplemental oxygen–free days (SOFDs) through day 28, defined as the number of days alive and not receiving supplemental oxygen from the time of hospital presentation to day 28.^31^ Patients receiving the same amount of supplemental oxygen as chronic home oxygen until admission were considered free from supplemental oxygen. Patients with in-hospital death before day 28 received a value of −1 SOFD, consistent with previous literature defining death as worse than prolonged ventilation or receipt of supplemental oxygen.^31,32^ Outcome assessment ceased at hospital discharge or day 28, whichever occurred first. Patients discharged from the hospital prior to day 28 were presumed to be maintained at the supplemental oxygen level they were receiving at hospital discharge. Days when patients were intubated and ventilated exclusively for a surgical procedure and extubated immediately on completion of the procedure did not count toward the SOFD calculation. SOFD has been validated as an outcome in clinical trials^31^ and used as the primary outcome in trials of acutely ill patients.^33^ Specifically, SOFDs extend the well-known concept of ventilator-free days (VFDs) into a larger, less severely ill population of patients who may or may not receive invasive mechanical ventilation.

We assessed safety via hypoxemia (defined as Spo_2_ <88%) during the ICU stay, in-hospital mortality, and adverse events (defined as any unintended adverse consequences of targeting normoxemia). Prespecified secondary outcomes were assessed at 28 days or 90 days or until hospital discharge, if sooner. Key secondary outcomes included VFDs to day 28,^34^ in-hospital mortality to day 90, and hospital-free days (HFDs) to day 90.^27^ We also completed prespecified subgroup analyses to assess the heterogeneity of treatment effect. These subgroups included penetrating vs nonpenetrating trauma, traumatic brain injury, receipt of mechanical ventilation before ICU admission, presence of shock, and high (≥16) vs low (<16) Injury Severity Score (ISS; median score [IQR] range: 0-75 [10-26], with higher scores indicating more severe injury).^27^

### Statistical Analysis

Details of the sample size calculation have been reported elsewhere.^27^ Using data from the pilot study,^26^ we estimated that during the 28 months of the trial, at least 6000 patients would be enrolled and included in the primary analysis. We estimated the mean (SD) SOFDs would be 15.5 (11.3), and the intraclass correlation coefficient would be 0.04. We calculated that enrolling 6000 patients would allow us to detect a difference in SOFDs of 1.42 days at 80% power and 1.64 days at 90% power, with α = .005.

The primary analysis compared the number of SOFDs among the targeted normoxemia group vs usual care group using linear mixed-effects modeling (LMM). We included a fixed effect for time to account for possible temporal trends associated with intervention implementation at different times as well as a random intercept to account for the clustering of patients within sites. We used a *t* distribution with 6 *df* for hypothesis testing and CI calculation to account for the small number of clusters in the sample. Because we anticipated some imbalances in baseline characteristics in the stepped-wedge trial design, we adjusted the model for age, sex, race and ethnicity, body mass index, tobacco use, insurance type, number of Elixhauser comorbidities,^35^ mechanism of injury, ISS, and mechanical ventilation status at ICU admission. Race and ethnicity were self-reported by patients (Hispanic, non-Hispanic Black [hereafter Black], non-Hispanic White [hereafter White], other [including American Indian or Alaska Native, Asian, and multiracial], or unknown) and were collected because skin pigmentation may affect pulse oximetry performance. Due to data-completeness issues with post-ICU oxygen data at 1 site, we applied a multiple imputation approach using data from that site and other sites for the primary outcome analysis. Five imputed datasets were generated, with the imputation model being used only for the subset of missing SOFD values at this site. The LMM was then estimated separately on each imputed dataset, and then results were combined using the standard formulae.^36^

We also performed sensitivity analyses for the primary outcome wherein the site with data-completeness issues was removed entirely and only ICU data (which were complete for all sites) were included. Other sensitivity analyses examined model assumptions for the primary outcome.

Based on the pilot study, we expected a small amount of oxygenation data not affecting the primary outcome to be missing due to charting inconsistencies. To account for missing data, we assumed that for patients receiving mechanical ventilation, FIo_2_ remained constant until a patient was extubated or a new FIo_2_ was entered. For patients not receiving mechanical ventilation, we assumed that supplemental oxygen flow rate remained constant for up to 12 hours until a new value was entered. After 12 hours without a new measurement recorded, the patient was assumed to be on room air (ie, receiving no supplemental oxygen). Missing covariate data were handled by adding an explicit level for *missing* to the existing levels for each variable.

We analyzed continuous secondary outcomes (ie, VFDs to day 28 and HFDs to day 90) using an LMM approach similar to that used for the primary outcome. We used a mixed-effects logistic regression model for dichotomous outcomes and a mixed-effects ordinal logistic regression model for ordinal outcomes (eg, GOS score). For time-to-event outcomes (ie, time to room air, time to mortality), we used a Cox proportional hazards regression model with a γ-distributed frailty for site. We adjusted all secondary outcome regression models for time and for all patient-level covariates adjusted for in the primary outcome analysis.

All statistical analyses were based on the intention-to-treat approach and performed from April 2023 to November 2024 using R, version 4.2.2 (R Core Team).^37^ We performed estimation of Cox proportional hazards regression models using the survival package in R.^38^ Two-sided *P* = .05 indicated statistical significance.

## Results

**Figure 1.**
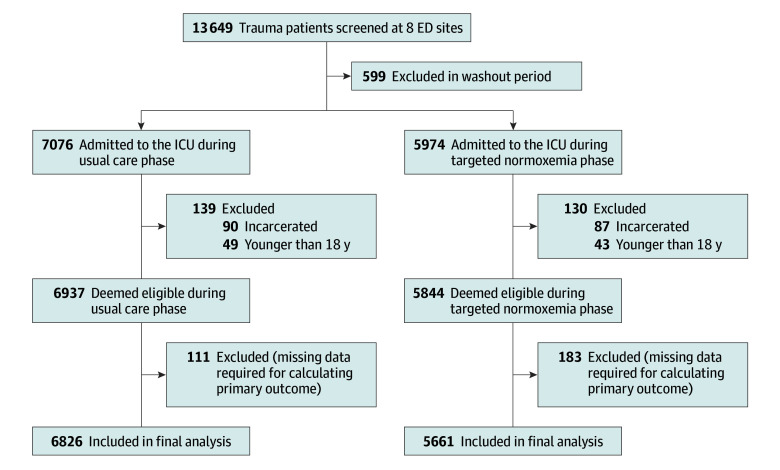
Study Flow Diagram Enrollment of patients was based on prespecified inclusion and exclusion criteria. A total of 294 patients were excluded after eligibility was determined due to missing data, which made analysis of the primary outcome (supplemental oxygen–free days) impossible. ED indicates emergency department; ICU, intensive care unit.

Among 12 487 critically ill trauma patients (5661 in the targeted normoxemia group and 6826 in the usual care group) enrolled in the trial, the mean (SD) age was 51.7 (21.1) years; of these participants, 3688 were females (29.5%) and 8799 were males (70.5%); 1971 identified as Black (15.8%), 1125 as Hispanic (9.0%), 7018 as White (56.2%), 889 as other (7.1%), and 1484 had unknown (11.9%) race and ethnicity; and the mean (SD) ISS was 19.6 (12.0) (Table 1 and Figure 1). Imbalances in baseline characteristics included race and ethnicity, incidence of traumatic brain injury, penetrating mechanism of injury, and mechanical ventilation status before ICU admission (Table 1; eTable 1 in Supplement 2). Missing data for most patient characteristics and outcomes were similar between groups (eTable 2 in Supplement 2).

**Table 1.** Patient Demographics and Injury Characteristics at Baseline

Characteristic	Patients, No. (%)	
	Targeted normoxemia group (n = 5661)	Usual care group (n = 6826)
Age, mean (SD), y	53.2 (21.3)	50.4 (20.9)
Sex		
Female	1655 (29.2)	2033 (29.8)
Male	4006 (70.8)	4793 (70.2)
Race and ethnicity^a^		
Hispanic	642 (11.3)	483 (7.1)
Non-Hispanic Black	599 (10.6)	1372 (20.1)
Non-Hispanic White	2930 (51.8)	4088 (59.9)
Other^b^	376 (6.6)	513 (7.5)
Unknown	1114 (19.7)	370 (5.4)
BMI, mean (SD)	27.9 (6.6)	28.2 (6.9)
Current or former smoker	1212 (21.4)	1305 (19.1)
Supplemental oxygen use at baseline	76 (1.3)	127 (1.9)
No. of Elixhauser comorbidities, mean (SD)	2.9 (2.3)	2.4 (2.2)
Cardiac comorbidities	882 (15.6)	1130 (16.6)
Pulmonary comorbidities	494 (8.7)	720 (10.5)
Penetrating mechanism of injury^c^	699 (12.3)	1161 (17.0)
EMS mode of arrival	5391 (95.2)	6616 (96.9)
Initial GCS score, mean (SD)	12.1 (4.3)	11.7 (4.5)
ISS, mean (SD)	19.3 (11.7)	19.9 (12.2)
TBI	2616 (46.2)	1879 (27.5)
Mechanical ventilation before ICU	1846 (32.6)	2826 (41.4)
Proportion of time receiving invasive mechanical ventilation, mean (SD), %	22.3 (34.2)	26.2 (34.5)
Mechanical ventilation at any time during ICU admission	2321 (41.0)	3388 (49.6)

Abbreviations: BMI, body mass index (calculated as weight in kilograms divided by height in meters squared); EMS, emergency medical service; GCS, Glasgow Coma Scale (score range: 3-15, with the highest score indicating a fully awake, alert, and oriented patient); ICU, intensive care unit; ISS, Injury Severity Score (median score [IQR] range: 0-75 [10-26], with higher scores indicating more severe injury); TBI, traumatic brain injury.

^a^ Race and ethnicity were self-reported by patients and obtained from each site’s electronic health record.

^b^ Other included American Indian or Alaska Native, Asian, and multiracial.

^c^ Injury classifications: penetrating includes any stabbing, cut, or gunshot wounds.

### Oxygenation in ICU

**Figure 2.**
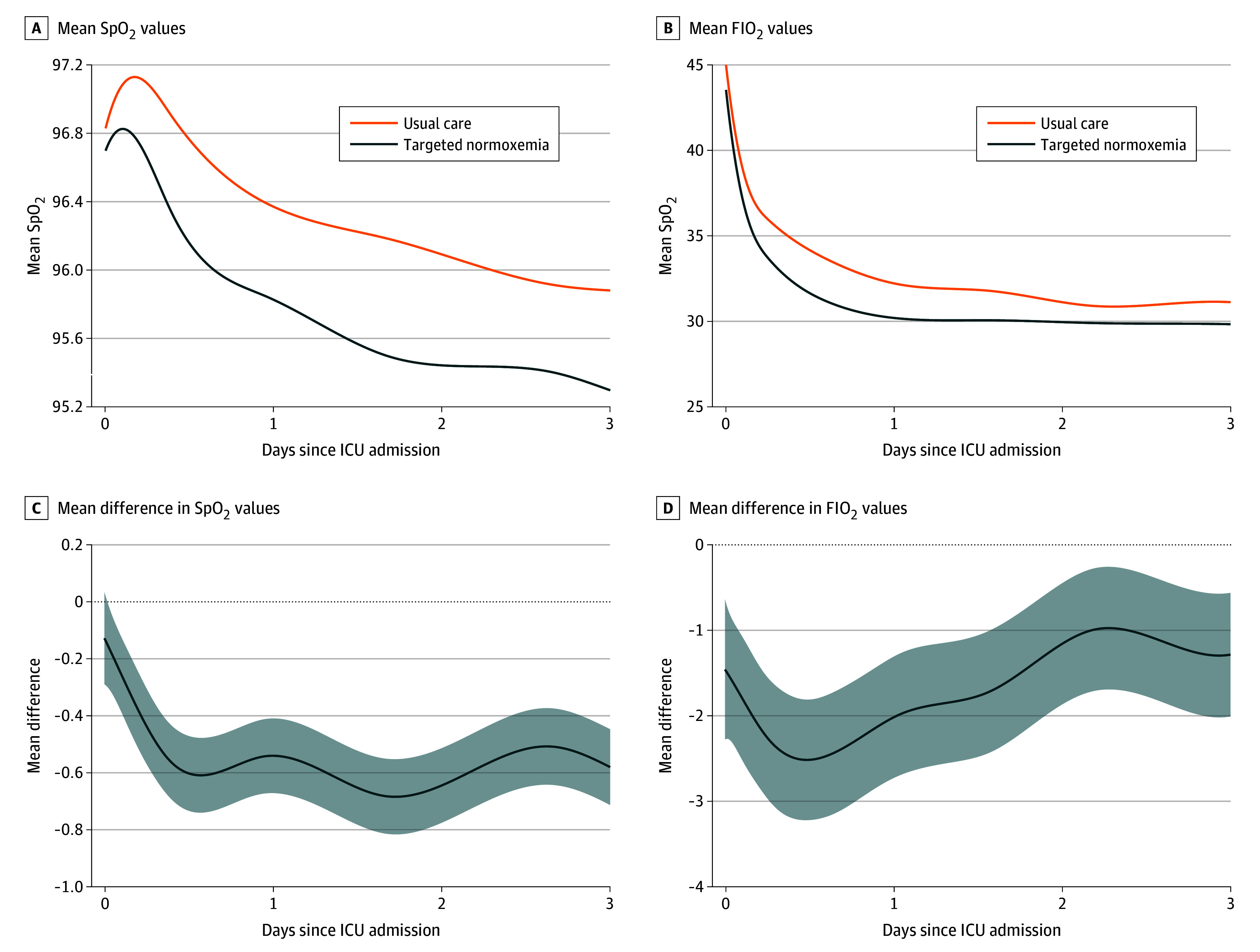
Mean FIo_2_ and Oxygen Saturation Measured by Pulse Oximetry (Spo_2_) Values During Intensive Care Unit (ICU) Admission for Modifiable Patient Time Shaded areas represent 95% CIs. Patient time was considered nonmodifiable if the patient was receiving FIo_2_ of 21% (ie, room air) but had Spo_2_ greater than 96% (ie, hyperoxemia range). All other patient time is included.

We measured 2 160 042 Spo_2_ values between enrollment and ICU discharge among the 12 487 participants. The median (IQR) number of Spo_2_ measurements per patient was 100 (48-206). The proportion of ICU time spent in normoxemia increased from 56.2% in the usual care group to 71.6% in the targeted normoxemia group. Hyperoxemia with an FIo_2_ greater than 21% decreased from 42.4% in the usual care group to 26.7% in the targeted normoxemia group, while hypoxemia was similar between groups (1.1% vs 1.1%) (Table 2). The mean FIo_2_ and Spo_2_ values during the first 3 days of ICU admission are displayed in Figure 2 along with the mean difference between intervention conditions (targeted normoxemia minus usual care). These patterns were similar when analyzing only modifiable patient time (eFigure 7 in Supplement 2) and all patient time in the hospital (eFigure 8 in Supplement 2). The proportion of patient time spent with an FIo_2_ of 21% (room air) increased from 37.9% in the usual care group to 55.2% in the targeted normoxemia group. The mean (SD) total volume of oxygen administered per patient (32 565 [62 793] L vs 18 862 [43 097] L; adjusted mean difference [AMD], −5500 [95% CI, −8720 to −2280] L) and the mean (SD) oxygen flow rate (3.3 [3.7] L/min vs 2.2 [3.3] L/min; AMD, −0.54 [95% CI, −0.72 to −0.35] L/min) also decreased from the usual care group to the targeted normoxemia group (Table 2). We observed similar patterns for FIo_2_ and Spo_2_ when stratified by race and ethnicity (eFigure 6 in Supplement 2).

**Table 2.** Primary and Main Secondary Outcomes

Outcome	Mean (SD)		Adjusted mean difference (95% CI)
	Targeted normoxemia group (n = 5661)	Usual care group (n = 6826)	
Primary outcome: SOFDs through day 28	19.6 (10.3)	17.5 (10.4)	0.32 (−0.37 to 1.00)
*P* value	NA	NA	.30
In-hospital mortality to day 90, No. (%)	563 (9.9)	732 (10.7)	AHR: 1.05 (0.83 to 1.33)^a^
HFDs through day 90	69.8 (27.4)	69.0 (27.5)	1.16 (−0.35 to 2.68)
Hospital LOS, d	13.1 (17.4)	13.2 (16.1)	AHR: 1.08 (0.99 to 1.18)^a^
ICU LOS, d	5.7 (7.7)	6.3 (10.3)	−0.03 (−0.09 to 0.03)
VFDs through day 28^b^	23.3 (9.2)	22.4 (9.7)	0.55 (0.03 to 1.08)
Time to room air, d	1.6 (3.2)	2.7 (4.0)	AHR: 1.23 (1.13 to 1.33)^a^
Total volume of oxygen administered per patient, L	18 862 (43 097)	32 565 (62 793)	−5500 (−8720 to −2280)
Total volume of oxygen administered per patient, L/min	2.2 (3.3)	3.3 (3.7)	−0.54 (−0.72 to −0.35)
Proportion of time spent in normoxemia (Spo_2_ 90%-96%) in ICU, %	0.72 (0.29)	0.56 (0.32)	0.07 (0.06 to 0.09)
Proportion of time spent in hyperoxemia (Spo_2_ >96%) in ICU, %	0.27 (0.29)	0.42 (0.31)	−0.07 (−0.09 to −0.06)
Proportion of time spent in hypoxemia (Spo_2_ <88%) in ICU, %	0.011 (0.06)	0.011 (0.06)	0.0001 (−0.004 to 0.004)
Discharge disposition	NA	NA	AOR: 1.02 (0.89 to 1.17)^c^
Death or hospice, No. (%)	613 (10.8)	706 (10.3)	NA
Facility, No. (%)	1679 (29.7)	2229 (32.7)	NA
Home, No. (%)	3256 (57.5)	3672 (53.8)	NA

Abbreviations: AHR, adjusted hazard ratio; AOR, adjusted odds ratio; HFDs, hospital-free days; ICU, intensive care unit; LOS, length of stay; NA, not applicable; SOFDs, supplemental oxygen–free days; Spo_2_, oxygen saturation measured by pulse oximetry; VFDs, ventilator-free days.

^a^AHR for in-hospital 90-day mortality is greater than 1 nonsignificantly, indicating that targeted normoxemia had a mortality outcome similar to that in usual care. However, an AHR greater than 1 for hospital LOS until being discharged alive (benefit outcome) and receiving room air (benefit outcomes) suggests that targeted normoxemia offered greater benefits compared with usual care.

^b^ VFDs were not available for analysis at 1 of the 8 sites. This was the same site with post-ICU oxygen data completeness issues.

^c^Cumulative AOR greater than 1 means the targeted normoxemia group had a healthier discharge status or disposition (bottom categories) than usual care.

### Primary Outcome

**Figure 3.**
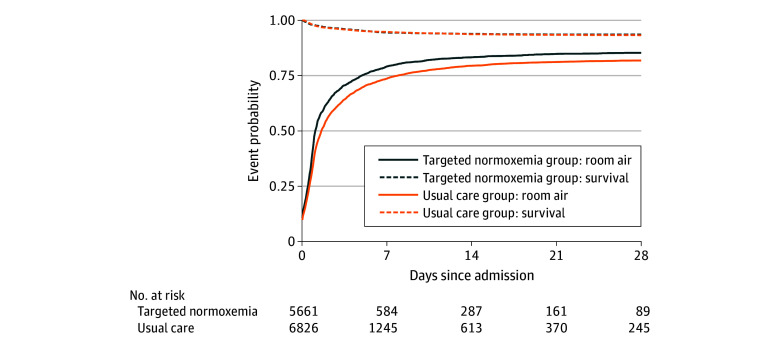
Proportion of Patients Alive and Not Receiving Supplemental Oxygen Through Day 28, by Intervention Group The top curve is a Kaplan-Meier mortality plot. The bottom curve is time to room air (ie, receiving no supplemental oxygen).

The raw mean (SD) SOFD through day 28 was greater in the targeted normoxemia group than in the usual care group (19.6 [10.3] vs 17.5 [10.4] days). Comparing targeted normoxemia to usual care, the AMD was 0.32 days (95% CI, −0.37 to 1.00 days; *P* = .30) (Table 2 and Figure 3). The intraclass correlation coefficient was estimated to be 0.07. The proportion of patients in each quartile of SOFD is displayed in eFigure 9 in Supplement 2.

### Secondary Outcomes

In-hospital mortality to day 90 occurred in 563 patients (9.9%) in the targeted normoxemia group and 732 patients (10.7%) in the usual care group (adjusted hazard ratio [AHR], 1.05; 95% CI, 0.83-1.33) (Table 2 and Figure 3; AHR greater than 1 was not statistically significant, indicating that targeted normoxemia had a mortality outcome similar to that in usual care. The raw mean (SD) HFD through day 90 was 69.8 (27.4) days in the targeted normoxemia group and 69.0 (27.5) days in the usual care group (AMD, 1.16; 95% CI, −0.35 to 2.68 days). The raw mean (SD) VFD through day 28 was 23.3 (9.2) days in the targeted normoxemia group and 22.4 (9.7) days in the usual care group (AMD, 0.55; 95% CI, 0.03-1.08 days). Patients were weaned to room air or 21% FIo_2_ earlier in the targeted normoxemia vs usual care group (mean [SD] time, 1.6 [3.2] vs 2.7 [4.0] days; AHR, 1.23 [95% CI, 1.13-1.33] days) (Table 2). An AHR greater than 1 for hospital length of stay until being discharged alive (benefit outcome) and receiving room air (benefit outcomes) suggests that targeted normoxemia offered greater benefits compared with usual care. Other prespecified secondary outcomes, including ICU length of stay, discharge disposition, and GOS score (Table 2; eTable 3 in Supplement 2) were similar between groups.

### Subgroup Analyses

Among patients not receiving mechanical ventilation at ICU admission, the mean SOFD was greater in the targeted normoxemia group than in the usual care group (22.6 [8.30] vs 20.6 [8.86] days; AMD, 0.75; 95% CI, 0.00-1.50 days) (eFigure 3 in Supplement 2). The mean values for the primary outcome did not differ between targeted normoxemia and usual care in any of the other prespecified subgroups (eFigure 3 in Supplement 2).

### Sensitivity Analyses

We performed sensitivity analyses to assess the impact of missing data on the results. Using data from the 7 sites with complete data, we found that results were similar to the primary analysis: the raw mean (SD) SOFD through day 28 was 19.9 (10.3) days in the targeted normoxemia group and 17.8 (10.4) days in the usual care group (AMD, 0.34; 95% CI, −0.37 to 1.05 days; *P* = .29) (eFigure 4 and eTable 4 in Supplement 2). Data from the ICU, where the intervention occurred, were complete for all sites. Using only ICU data for all 8 sites, the AMD for SOFD was similar to that in the primary analysis (AMD, 0.60; 95% CI, 0.02-1.22 days). Using only ICU data for the 7 sites with complete hospital data, AMD for SOFD was also similar (AMD, 0.46; 95% CI, −0.19 to 1.11 days).

We also performed sensitivity analyses to assess the appropriateness of model assumptions. Without making the assumption of conditional normality for SOFD, we found results similar to the primary analysis using a mixed-effects proportional odds model (ie, treating SOFD as an ordinal variable with 30 levels). The adjusted cumulative odds ratio was 1.22 (95% CI, 1.02-1.46), reflecting a slight upward shift in the distribution of SOFD for the targeted normoxemia group compared with the usual care group. A marginal modeling approach (linear model adjusted for site as a fixed effect with robust variance estimators—that is, not assuming a mixed model) similarly found a slight positive treatment effect (AMD, 0.27; 95% CI, −0.74 to 1.28), which is also consistent with the primary analysis (eFigure 5 in Supplement 2). No adverse events were reported throughout the study period.

## Discussion

Among adult trauma patients admitted to a participating ICU, targeting normoxemia did not significantly increase the number of SOFD but did reduce the administration of supplemental oxygen without substantive increases in hypoxemia, death, or adverse events. There were no differences in 90-day in-hospital mortality or HFDs between groups, but we observed greater VFDs in the targeted normoxemia group. The clinical implication of these findings is that, among critically injured adults, targeting normoxemia can safely decrease use and expedite liberation from supplemental oxygen.

These results are consistent with those of a recent meta-analysis of 13 randomized clinical trials of oxygenation targets in critically ill patients,^21^ which extends the present findings to a novel population of critically injured adults. We exclusively enrolled critically ill trauma patients, who represented up to 14.5%^14^ of patients in previous trials focusing on medical illnesses. There was no difference in mean SOFDs when targeting normoxemia in a trauma-specific population overall. However, we observed an increase in SOFDs among patients not mechanically ventilated, an understudied population in prior trials focused on patients receiving mechanical ventilation.

Additionally, the results suggest that use of supplemental oxygen can be safely reduced by targeting normoxemia. Since oxygen supply is limited in military and other austere settings, safely reducing the use of supplemental oxygen in critically ill trauma patients has logistical importance. Furthermore, movement of equipment and supplies around a battlefield comes with additional challenges not encountered in the civilian setting; thus, improved logistics confer many advantages.^39,40^

### Strengths and Limitations

This trial has several strengths. The sample, to our knowledge, is the largest to date among trials comparing oxygenation targets, allowing for more precise estimates of treatment effect. Key subgroups were represented, including patients with traumatic brain injury.^23,41^ Findings from these subgroups may inform areas of future investigation. Furthermore, we were able to achieve immediate implementation of the intervention from patient arrival to the participating ED through the entire ICU stay. Enrolling patients from 8 geographically and demographically diverse sites and the pragmatic implementation in usual clinical practice enhanced the generalizability and clinical applicability of trial results. Specifically, embedding targeted normoxemia into usual clinical care across many diverse sites increased the external validity of the findings.

The findings should be interpreted in the context of several limitations. We selected a stepped-wedge, cluster-randomized design rather than patient-level randomization to improve generalizability and clinical applicability.^42^ As a result, some baseline characteristics were unbalanced between groups, contributing to some differences between raw and adjusted results. However, this outcome is not unusual given the stepped-wedge design. Depending on when a site was randomized to crossover from usual care to targeted normoxemia, it would contribute proportionally more data to one phase over another, which highlights the importance of adjusting for patient-level characteristics in regression models, a key component of all analyses. In addition to adjusting for patient-level covariates and temporal trends, we also included site-specific random effects in all adjusted models to capture site heterogeneity. While including more than 8 clusters (sites) would likely have decreased imbalances between groups and increased the power of the trial, including additional sites would have been infeasible. A broad intervention among many hospital units, including the ED and ICU, is subject to an initial learning curve and ongoing maintenance challenges for the health care team. Although a 1-month run-in period was provided between the usual care and targeted normoxemia phases in order for clinical staff to master the implementation of targeted normoxemia, adherence to the protocol may have varied throughout the targeted normoxemia period at each site and reduced separation between groups for Spo_2_ and FIo_2_ (Figure 2). We did not have access to detailed information on any site-specific policy changes that may have occurred. However, we observed an overall increase in the proportion of patients weaned to room air on day 0 (Figure 3), suggesting that we achieved early changes in oxygen downtitration, starting on ED arrival. While achieving 100% of patient time in normoxemia among the targeted normoxemia group would have been ideal, it was impractical in this pragmatic trial. In addition, conduct of this trial during peak periods of the COVID-19 pandemic may have altered the implementation of the intervention and the associated results. Each site had their own COVID-19–related protocols, although the trial protocol guided oxygen titration at all sites. We also encountered data-completeness issues with post-ICU oxygen data at 1 site. To address these issues, we applied a multiple imputation approach using data from that site and other sites for the primary outcome analysis and performed multiple analyses to confirm the results of the primary analysis (eTable 3 and eFigure 4 in Supplement 2). In this trial, we assessed patients only through in-hospital outcomes through day 90; therefore, postdischarge and long-term outcomes remain unknown. The trial protocol did not control additional interventions, such as ventilator management; however, these interventions were standardized according to the institutional protocols at each site.

## Conclusions

In this randomized clinical trial, targeting normoxemia did not increase the number of SOFDs among critically ill trauma patients, but it safely reduced supplemental oxygen use without an associated increase in hypoxemia or adverse events. Targeting normoxemia appeared to increase the number of SOFD among critically ill trauma patients not receiving mechanical ventilation.

## Notes

Supplement 1.
Trial Protocol

Supplement 2.
eMethods
**eTable 1.** Additional Patient Demographics and Characteristics at Baseline **eTable 2.** Summary of Missing Patient Characteristics and Outcomes **eTable 3.** Oxygenation Outcomes and Additional Secondary Outcomes **eTable 4.** Sensitivity Analyses of Primary and Main Secondary Outcomes **eFigure 1.** Electronic Health Record Best Practice Alert **eFigure 2.** Multimodal Educational and Informatics Intervention Schematic **eFigure 3.** Heterogeneity of Treatment Effect for Supplemental Oxygen Free Days (SOFD) **eFigure 4.** Sensitivity Analysis of Heterogeneity of Treatment Effect for Supplemental Oxygen-Free Days (SOFD) Excluding the One Site With Data Completeness Issues **eFigure 5.** Sensitivity Analysis of Primary Outcome Model Specifications **eFigure 6.** SpO2 and FiO2 Stratified by Race/Ethnicity and Treatment Arm **eFigure 7.** A) Density of Patient Time Spent at Fraction of Inspired Oxygen (FiO2) and Oxygen Saturation (SpO2) by Group (Targeted Normoxemia vs. Usual Care) for Modifiable Patient Time; B) Change in the Density of Patient Time (Targeted Normoxemia Minus Usual Care) Spent at FiO2 and SpO2 for Modifiable Patient Time **eFigure 8.** A) Density of Patient Time Spent at Fraction of Inspired Oxygen (FiO2) and Oxygen Saturation (SpO2) by Group (Targeted Normoxemia vs Usual Care) for All Patient Time; B) Change in the Density of Patient Time (Targeted Normoxemia Minus Usual Care) Spent at FiO2 and SpO2 for All Patient Time **eFigure 9.** Proportion of Patients by Quartile of Supplemental Oxygen-Free Days
eReferences

Supplement 3.
Nonauthor Collaborators

Supplement 4.
Data Sharing Statement
