# Supplementary material for: Targeted Normoxemia and Supplemental Oxygen–Free Days in Critically Injured Adults: A Stepped-Wedge Cluster Randomized Clinical Trial
Source: JAMA Netw Open. 2025 Mar 31;8(3):e252093. doi: 10.1001/jamanetworkopen.2025.2093 (PMC12186824; doi:10.1001/jamanetworkopen.2025.2093)
Supplement: Supplement 2. — eMethods eTable 1. Additional Patient Demographics and Characteristics at Baseline eTable 2. Summary of Missing Patient Characteristics and Outcomes eTable 3. Oxygenation Outcomes and Additional Secondary Outcomes eTable 4. Sensitivity Analyses of Primary and Main Secondary Outcomes eFigure 1. Electronic Health Record Best Practice Alert eFigure 2. Multimodal Educational and Informatics Intervention Schematic eFigure 3. Heterogeneity of Treatment Effect for Supplemental Oxygen Free Days (SOFD) eFigure 4. Sensitivity Analysis of Heterogeneity of Treatment Effect for Supplemental Oxygen-Free Days (SOFD) Excluding the One Site With Data Completeness Issues eFigure 5. Sensitivity Analysis of Primary Outcome Model Specifications eFigure 6. SpO2 and FiO2 Stratified by Race/Ethnicity and Treatment Arm eFigure 7. A) Density of Patient Time Spent at Fraction of Inspired Oxygen (FiO2) and Oxygen Saturation (SpO2) by Group (Targeted Normoxemia vs. Usual Care) for Modifiable Patient Time; B) Change in the Density of Patient Time (Targeted Normoxemia Minus Usual Care) Spent at FiO2 and SpO2 for Modifiable Patient Time eFigure 8. A) Density of Patient Time Spent at Fraction of Inspired Oxygen (FiO2) and Oxygen Saturation (SpO2) by Group (Targeted Normoxemia vs Usual Care) for All Patient Time; B) Change in the Density of Patient Time (Targeted Normoxemia Minus Usual Care) Spent at FiO2 and SpO2 for All Patient Time eFigure 9. Proportion of Patients by Quartile of Supplemental Oxygen-Free Days eReferences [file jamanetwopen-e252093-s002.pdf]

Supplemental Online Content

Douin DJ, Rice JD, Anderson EL, et al; for the Strategy to Avoid Excessive Oxygen (SAVE-O2) Investigators. Targeted normoxemia and supplemental oxygen–free days in critically injured adults: a stepped-wedge cluster randomized clinical trial. *JAMA Netw Open*. Published online March 27, 2025. doi:10.1001/jamanetworkopen.2025.2093

eMethods.

eTable 1. Additional Patient Demographics and Characteristics at Baseline

eTable 2. Summary of Missing Patient Characteristics and Outcomes

eTable 3. Oxygenation Outcomes and Additional Secondary Outcomes

eTable 4. Sensitivity Analyses of Primary and Main Secondary Outcomes

eFigure 1. Electronic Health Record Best Practice Alert

eFigure 2. Multimodal Educational and Informatics Intervention Schematic

eFigure 3. Heterogeneity of Treatment Effect for Supplemental Oxygen Free Days (SOFD)

eFigure 4. Sensitivity Analysis of Heterogeneity of Treatment Effect for Supplemental Oxygen-Free Days (SOFD) Excluding the One Site With Data Completeness Issues

eFigure 5. Sensitivity Analysis of Primary Outcome Model Specifications

eFigure 6. SpO2 and FiO2 Stratified by Race/Ethnicity and Treatment Arm

eFigure 7. A) Density of Patient Time Spent at Fraction of Inspired Oxygen (FiO2) and Oxygen Saturation (SpO2) by Group (Targeted Normoxemia vs. Usual Care) for Modifiable Patient Time; B) Change in the Density of Patient Time (Targeted Normoxemia Minus Usual Care) Spent at FiO2 and SpO2 for Modifiable Patient Time

eFigure 8. A) Density of Patient Time Spent at Fraction of Inspired Oxygen (FiO2) and Oxygen Saturation (SpO2) by Group (Targeted Normoxemia vs. Usual Care) for All Patient Time; B) Change in the Density of Patient Time (Targeted Normoxemia Minus Usual Care) Spent at FiO2 and SpO2 for All Patient Time

© 2025 Douin DJ et al. *JAMA Network Open*

eFigure 9. Proportion of Patients by Quartile of Supplemental Oxygen-Free Days

eReferences

This supplemental material has been provided by the authors to give readers additional information about their work.

© 2025 Douin DJ et al. *JAMA Network Open*

eMethods

IRB Approval and Waiver of Informed Consent

Trauma patients admitted to an intensive care unit (ICU) are at significant risk for morbidity and mortality from their underlying illness. Most critically ill trauma patients receive supplemental oxygen with either invasive mechanical ventilation or various other methods of oxygen delivery (noninvasive ventilation, high-flow nasal oxygen, oxygen mask, nasal cannula, etc.). Oxygen therapy has undisputed importance in the care of these patients to prevent morbidity associated with hypoxemia and enhance oxygen delivery. However, excessive oxygen supplementation resulting in hyperoxemia in critically ill patients is routine and may be harmful.

Prior to the design of the SAVE-O2 trial, we conducted a systematic review,[1] Delphi consensus process,[2] and single-center pilot trial.[3] Through these processes, we demonstrated preliminary safety evidence for targeting normoxemia (SpO2 90-96% or PaO2 60-100mmHg) and identified consensus-based oxygenation targets. These data provided a foundation to conduct our multicenter clinical trial with a waiver of informed consent under the Common Rule (45 CFR 46). Specifically, the trial intervention, a multimodal educational and informatics intervention to target normoxemia, represented no more than minimal risk to participants. Further, oxygenation targets were not binding and remained at the discretion of the treating physician to ensure optimal care of all critically ill trauma patients. Waiver of informed consent did not adversely affect the rights or welfare of participants, and patient care remained at the discretion of the clinical team to act in the best interest of each patient. Further, data was collected at the hospital-unit level to ensure patient privacy was not compromised. Finally, the study could not practically be carried out without a waiver of consent because there was no direct contact between research personnel and study participants or their surrogates. The intervention

© 2025 Douin DJ et al. *JAMA Network Open*

was conducted at the level of each hospital unit, with participants as a whole receiving either usual care in the pre-intervention period, or the multimodal intervention enhanced care targeting normoxemia in the post-intervention period.

Because the study involved minimal incremental risk, did not adversely affect the welfare or privacy rights of the participants, and because obtaining informed consent was impractical, a waiver of informed consent was requested from and approved by the Colorado Multiple Institutional Review Board (COMIRB #19-2153) which served as the single IRB for this study. Each enrolling site ceded review to COMIRB under reliance agreements. The trial was registered at ClinicalTrials.gov before initiation (NCT04534959) and overseen by an independent data and safety monitoring board.

Stepped Wedge Cluster Randomized Trial Design

The following schematic displays the randomized timing of crossover from usual care (pre-intervention) to targeted normoxemia (post-intervention) for the eight SAVE-O2 sites. Each hospital-unit represented a cluster, and cluster crossover occurred every three months. We randomly assigned the sequence when each cluster would crossover. The first crossover was initiated three months after the start of data collection, as the stepped wedge design requires one period during which all units remain in the control condition. The crossover included a one-month run-in period during which clinical staff engaged in educational activities and training designed to increase familiarity and compliance with targeted normoxemia protocols. Enrollment began on July 15, 2020, and concluded on November 14, 2022.

© 2025 Douin DJ et al. *JAMA Network Open*

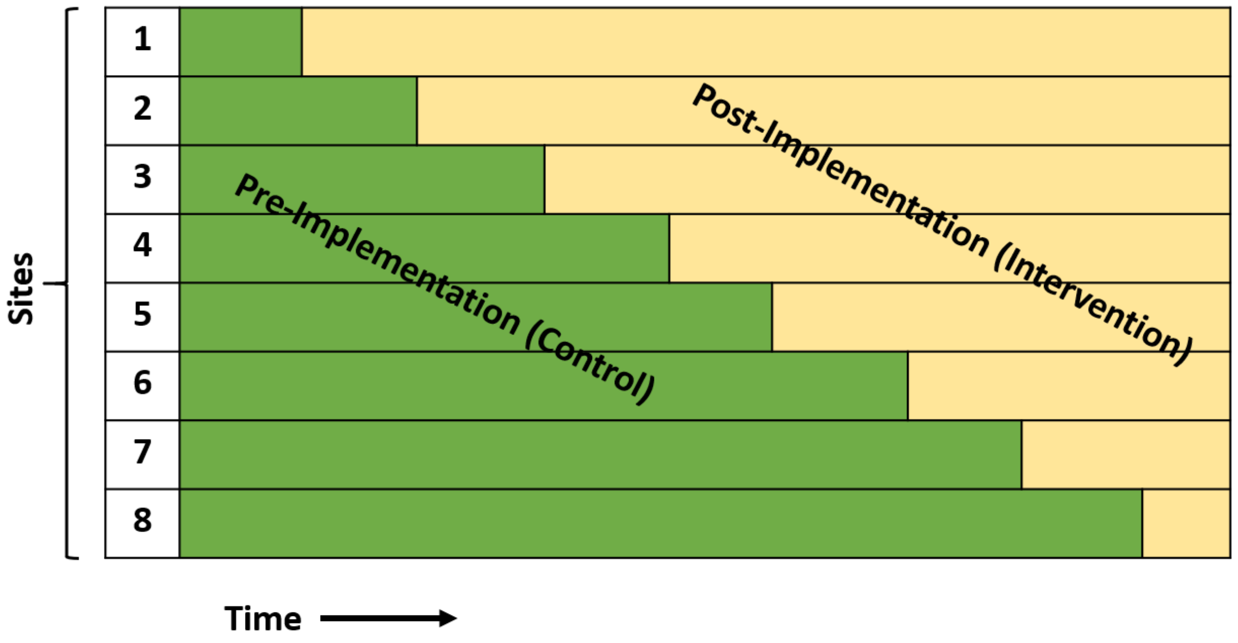

Inclusion and Exclusion Criteria

The inclusion criteria for the study were:

1. Acutely injured patients who meet the criteria for entry into the state or national trauma registry
2. Admission to surgical/trauma ICU within 24 hours of hospital arrival

The exclusion criteria for the study were:

1. Patient is known to be less than 18 years old
2. Patient is known to be pregnant
3. Patient is known to be a prisoner
4. Transferred patients not admitted through the emergency department

Multimodal Educational and Informatics Trial Intervention

Complete details of trial outcomes were published in the protocol and statistical analysis plan.[4] We conducted a multi-faceted approach to clinician and staff education at each of the eight participating sites. These included presentations tailored to specific provider groups – physicians, advanced practice providers, nurses, and respiratory therapists. We provided the background/rationale for the SAVE-O2 trial, detailed our oxygen titration protocol, and instructed sites on how to report potential adverse events. We also provided feedback and guidance during monthly investigator meetings.

Oxygenation Protocol

Our protocol provided non-binding recommendations for down-titration of the flow rate or supplemental oxygen of the FiO2 in patients with sustained hypoxemia (SpO2>96% or PaO2>100mmHg for ≥30 minutes) to achieve the target SpO2 (90-96%). Oxygen was down-titrated by FiO2 increments of 0.1 (for mechanically ventilated patients) or 1-2 liters per minute (for non-mechanically ventilated patients) until normoxemia was achieved or the patient reached an FiO2 of 0.21 or room air. Treating physicians were allowed to override study recommendations when they determined it was in the best interest of the patient. For example, in the setting of carbon monoxide poisoning, untreated pneumothorax, and suspected cyanide poisoning.

The trial protocol directed FiO2/oxygen flow rate adjustment to the assigned SpO2 target only during the patient's index ICU admission. After patients were discharged from the ICU or transferred to a floor bed, their oxygenation titration was no longer part of the trial protocol and was at the discretion of the clinical team.

© 2025 Douin DJ et al. *JAMA Network Open*

Data Collection

We extracted data on baseline characteristics, treatment received during the trial, and in-hospital outcomes from each site's electronic health records (EHRs) and state trauma registries. Each site entered data into their local instance of the REDCap software.[5] These data were automatically de-identified and sent to the REDCap instance at the data coordinating center at Vanderbilt University Medical Center through the REDCap application programming interface. Some sites also used REDCap's Clinical Data Interoperability Services to automatically extract demographics, laboratory results, vital signs, oxygenation, and condition data from their local EHRs.[6] Study data included recorded SpO2, PaO2, FiO2, and oxygen volume measurements. Supplemental oxygen flow rates for non-mechanically ventilated patients were converted to FiO2 values using the conventional prediction model.[7] That is, for every liter of oxygen delivered, the FiO2 increases by 3.5% above room air or 21%. Trial personnel who were unaware of group assignments collected some data manually from the electronic health record using standardized operating procedures, including home supplemental oxygen use, Glasgow Outcomes Scale (GOS) at discharge, and military status. Full data collection protocols have been elaborated previously.[8]

Trial Outcomes

Complete details of trial outcomes were published in the protocol and statistical analysis plan.[4]

Primary outcome is as follows:

- Supplemental Oxygen Free Days (SOFD). This was defined as the number of days alive and not receiving supplemental oxygen through day 28 of hospitalization. SOFD was

© 2025 Douin DJ et al. *JAMA Network Open*

censored at hospital discharge. The score ranged from -1 (death) to 28 days (no supplemental oxygen use). Time spent intubated and ventilated only for a surgical procedure was noted as counted to the patient was immediately extubated upon completion of the procedure.

- **Secondary outcomes are as follows:**
  - 90-day all-cause in-hospital mortality
  - Ventilator-free days (VFD) in the first day 28 days
  - Hospital-free days (HFD) in the first 90 days
  - Hospital length of stay in days
  - ICU length of stay in days
  - Glasgow Outcome Scale (GOS) at hospital discharge
  - Discharge disposition

Mortality, VFD, and HFD were handled similarly to SOFD, where patients who died during the outcome assessment period (28 days for VFD and 90 days for HFD and mortality) received a value of -1.[8]

Oxygenation outcomes are as follows:

- Proportion of time spent in normoxemia (SpO2 90-96%) in the first 28 days
- Proportion of time spent in hyperoxemia (SpO2 >96%) in the first 28 days
- Proportion of time spent in hypoxemia (SpO2 <88%) in the first 28 days
- Proportion of time receiving >4 liters/minute or FiO2 >40% in the first 28 days
- Proportion of time receiving no supplemental oxygen in the first 28 days
- Total volume of oxygen administered in the first 28 days

© 2025 Douin DJ et al. *JAMA Network Open*

- Time to room air in days

Adverse events, defined as any unintended negative consequences of targeting normoxemia, were planned to be reported to the Department of Defense Scientific Officer, the Independent Safety Monitor, the Colorado Multiple Institutional Review Board (COMIRB), and the Defense Health Agency Office of Human and Animal Research Oversight in accordance with their reporting policies.

Sample Size Calculation

Based on data from our single-center pilot trial, we estimated a mean SOFD of 15.5 days with a standard deviation of 11.3 days and an intraclass correlation coefficient of 0.04. Based on these assumptions, we determined enrollment of at least 6,000 patients would allow us to detect a difference of 1.42 days in the primary outcome (SOFD) at 80% power and 1.64 days at 90% power. A difference of this magnitude would be considered clinically meaningful within the context of our trial.

We assumed a normal distribution of data and that each site would contribute an equal number of patients to the final analysis. Since our pilot data indicated that some of these assumptions may not be true (i.e., SOFD was restricted to a specific range, skewed, or bimodal), we conducted multiple simulation studies to address the impact of their violation on power. The simulation studies demonstrated almost no difference in power estimates relative to traditional power calculations. Further details of our sample size and power calculations have been previously reported.[8]

© 2025 Douin DJ et al. *JAMA Network Open*

Analysis of Primary Outcome

We employed a linear mixed effects modeling framework to analyze the primary outcome, SOFD. We included a fixed effect for time period to account for possible temporal trends associated with intervention implementation at different times, as well as a random intercept to account for the clustering of patients within sites. We accounted for clustering of patients within sites by including a random intercept term in all models. We also adjusted the model for the following patient-level covariates: age, sex, race/ethnicity, body mass index, tobacco use, insurance type, number of Elixhauser comorbidities,[9] mechanism of injury, injury severity score, and mechanical ventilation status at ICU admission.

Imbalances in baseline characteristics included race/ethnicity, incidence of traumatic brain injury, penetrating mechanism of injury, and mechanical ventilation status before ICU admission. However, this is not unusual given the stepped-wedge design of the trial: depending on when a site was randomized to cross over, it would contribute proportionally more data to one phase or the other. For example, a large site with a high proportion of Black patients being randomized to cross over later would result in an increase in the proportion of Black patients in the pre-intervention phase. This highlights the importance of adjusting for patient-level characteristics in regression models, a key component of all our analyses. In addition to adjusting for patient-level covariates, we also included site-specific random effects in all of our adjusted models to capture site heterogeneity.

Due to data completeness issues with post-ICU oxygen data at one site, we applied a multiple imputation approach using data from that site and other sites for the primary outcome analysis. Seven predicted values for the total number of oxygen days (up to 28 days) were generated per patient from seven right truncated regression models (one for each site with

© 2025 Douin DJ et al. *JAMA Network Open*

complete data). The truncated regression models included an adjustment for ICU oxygen days, ICU length of stay, hospital length of stay, ventilation on admission, and average FiO2 during the last six hours of ICU stay. For patients from the site with missing non-ICU data, a mean predicted outcome using all seven models was generated for each patient along with an average sigma value across all models. Those values were used to randomly generate five outcomes (number of oxygen days) per patient using the rtmvnorm package in R. To calculate SOFD, each of the five randomly generated total oxygen day values were subtracted from 28. However, if the predicted value was greater than the hospital length of stay, 28 was subtracted from the hospital length of stay. Similarly, if the predicted value was less than the total number of ICU oxygen days, 28 was subtracted from the total number of ICU oxygen days. Additionally, the following patients did not require an imputed outcome: patients who died (assigned a value of -1), patients who had more than 28 ICU days (as no more data was needed) and patients who were discharged from the hospital and ICU on the same day (no more data was needed). Five imputed data sets were generated, with the imputation model being used only for the subset of missing SOFD values at this site. Within each of the five imputed datasets, we obtained an estimate of the intervention effect on SOFD from a mixed model. Then, we applied Rubin's rule to pool effect estimates and their standard errors from five imputed datasets. The assumption is that the coefficient estimates of the intervention group follow a normal distribution, which is true in the mixed model setting. We used the pooled effect estimate and standard error to calculate 95% confidence intervals based on a t-distribution with a degree of freedom of 6. The linear mixed model was then estimated separately on each imputed data set and then results were combined using the standard formulae.[10] We also performed two sensitivity analyses. One where the site

with data completeness issues was removed entirely, and a second using only data from the ICU, where the targeted normoxemia intervention occurred, for all eight sites.

Secondary Analyses

We analyzed continuous secondary outcomes (i.e., ventilator-free days to day 28 and hospital-free days to day 90) using a linear mixed modeling approach similar to the primary outcome. We employed a logistic mixed model for dichotomous outcomes and a mixed-effects ordinal logistic regression for ordinal outcomes (e.g., Glasgow outcome scale). For time-to-event outcomes (i.e., time to room air, time to mortality), we used a Cox proportional hazards regression model with a gamma-distributed frailty intercept for site. Time zero was the patient's admission time. We adjusted all regression models for time period as well as all patient-level covariates adjusted for in the primary outcome analysis. All analyses were performed in R (Vienna, Austria).[11] We performed an estimation of all Cox models using the *survival* package.[12]

Handling of Missing Data

Based on our pilot study, we expected a small amount of oxygenation data would be missing due to charting inconsistencies. To account for missing data, we assumed that for mechanically ventilated patients, FiO2 remained constant until a patient was extubated or a new FiO_2 was entered. For non-mechanically ventilated patients, we assumed that delivered supplemental oxygen remained constant until a new value was entered up to 12 hours later. After 12 hours without a new measurement recorded, the patient was assumed to be on room air (receiving no supplemental oxygen).

© 2025 Douin DJ et al. *JAMA Network Open*

eTable 1. Additional Patient Demographics and Characteristics at Baseline

Characteristics	Targeted Normoxemia n=5,661	Usual Care n=6,826
Insurance Type, n (%)		
Private	1,482 (26.2)	1,845 (27.0)
Medicaid	1,557 (27.5)	1,859 (27.2)
Medicare	1,188 (21.0)	1,341 (19.6)
Uninsured	539 (9.5)	752 (11.0)
Other	652 (11.5)	870 (12.7)

© 2025 Douin DJ et al. *JAMA Network Open*

eTable 2. Summary of Missing Patient Characteristics and Outcomes

Outcome, n (%)	Targeted Normoxemia n=5,661	Usual Care n=6,826
Sex	5 (0.1%)	5 (0.1%)
Race/Ethnicity	1,114 (19.7%)	370 (5.4%)
Insurance Type	243 (4.3%)	159 (2.3%)
Current or Former Smoker	1,201 (21.2%)	1,514 (22.2%)
BMI	2,290 (40.5%)	2,302 (33.7%)
Number of Comorbidities	1,263 (22.3%)	1,153 (16.9%)
Mechanism of Injury	27 (0.5%)	11 (0.2%)
Initial Glasgow Comas Scale Score	40 (0.7%)	21 (0.3%)
Traumatic Brain Injury	123 (2.2%)	501 (7.3%)
Injury Severity Score	16 (0.3%)	14 (0.2%)
Mode of Arrival	153 (2.7%)	70 (1.0%)
Ventilator-Free Days through Day 28	813 (11.9%)	475 (8.4%)
Intensive Care Unit Length of Stay	17 (0.2%)	19 (0.3%)
Glasgow Outcome Score at Hospital Discharge	17 (0.2%)	19 (0.3%)
Discharge Disposition	219 (3.2%)	113 (2.0%)
Total Volume of Oxygen Administered per patient, mean (SD), L	74 (1.3%)	74 (1.1%)
Total Volume of Oxygen Administered per patient, mean (SD), L/min	74 (1.3%)	74 (1.1%)
Proportion of time spent in Normoxemia (SpO2 90-96%) in ICU	92 (1.6%)	120 (1.8%)
Received >4L/min or >40% FiO2 at any time during ICU Stay, n (%)	74 (1.3%)	74 (1.1%)
Proportion of time receiving >4L/min or >40% FiO2	74 (1.3%)	74 (1.1%)
Proportion of time spent in Hyperoxemia (SpO2 >96%) in ICU	92 (1.6%)	120 (1.8%)
Proportion of time spent in Hypoxemia (SpO2 <88%) in ICU	92 (1.6%)	120 (1.8%)

© 2025 Douin DJ et al. *JAMA Network Open*

eTable 3. Oxygenation Outcomes and Additional Secondary Outcomes

Outcome	Targeted Normoxemia n=5,661	Usual Care n=6,826	Adjusted Mean Difference (95% CI)
Received >4L/min or >40% FiO2 at any time during ICU Stay, n (%)	1,905 (33.7)	3,094 (45.3)	0.77[a] (0.66, 0.91)
Proportion of time receiving >4L/min or >40% FiO2	0.10 (0.22)	0.16 (0.26)	-0.04 (-0.06, -0.03)
Proportion of time receiving no supplemental oxygen or FiO2 21%	0.55 (0.41)	0.38 (0.39)	0.05 (0.03, 0.07)
SpO_2 During ICU Stay, mean (SD)	96.8 (3.4)	97.3 (3.4)	-0.29 (-0.41, -0.16)
FiO_2 During ICU Stay, mean (SD)	33.8 (16.3)	38.9 (17.0)	-1.85 (-2.51, -1.20)
Glasgow Outcome Scale at Hospital Discharge, n (%)			0.89[b] (0.78, 1.01)
Death	509 (9.0)	645 (9.4)	
Persistent Vegetative State	21 (0.4)	38 (0.6)	
Severe Disability	953 (16.8)	1,191 (17.4)	
Moderate Disability	1,590 (28.1)	1,715 (25.1)	
Low Disability	2,021 (35.7)	2,339 (34.3)	

FiO2, fraction of inspired oxygen; IQR, interquartile range; L, liters; min, minute; SpO2, pulse oximeter saturation. Note, Glasgow Outcome Scale at hospital discharge were not available for analysis at one of the eight sites. This was the same site with post-ICU oxygen data completeness issues.

Proportions of time reported here are only during patient time spent in the ICU

[a]Odds Ratio.

[b]Cumulative Odds Ratio. A cumulative Odds Ratio >1 means the targeted normoxemia group had a healthier discharge status or disposition (bottom categories) than usual care.

eTable 4. Sensitivity Analyses of Primary and Main Secondary Outcomes

Outcome	Targeted Normoxemia n=5,183	Usual Care n=6,013	Covariate-Adjusted Mean Difference (95% CI)
**Excluding one site with data completeness issues**			
**Primary Outcome:** Supplemental Oxygen Free Days through day 28, mean (SD)	19.9 (10.3)	17.8 (10.4)	0.34 (-0.24, 0.91) p=0.25
In-Hospital Mortality to day 90, n (%)	501 (9.7%)	626 (10.4%)	1.00[a] (0.74, 1.36)
Hospital-Free Days through day 90, mean (SD)	70.4 (27.2)	69.7 (27.4)	1.03 (-0.59, 2.64)
Hospital Length of Stay, mean (SD), d	12.7 (17.5)	12.8 (16.3)	1.09° (0.99, 1.19)

CI, confidence interval; d, days; ICU, intensive care unit; IQR, interquartile range; L, liters; min, minute; SD, standard deviation; SOFD, supplemental oxygen-free days.

[a]Hazard Ratio: The hazard ratio for in-hospital 90-day mortality is equal to 1, indicating that targeted normoxemia had a similar mortality outcome to usual care. However, a hazard ratio greater than 1 for hospital length of stay until being discharged alive (benefit outcome) suggests that targeted normoxemia offered greater benefits compared to usual care.

eFigure 1. Electronic Health Record Best Practice Alert

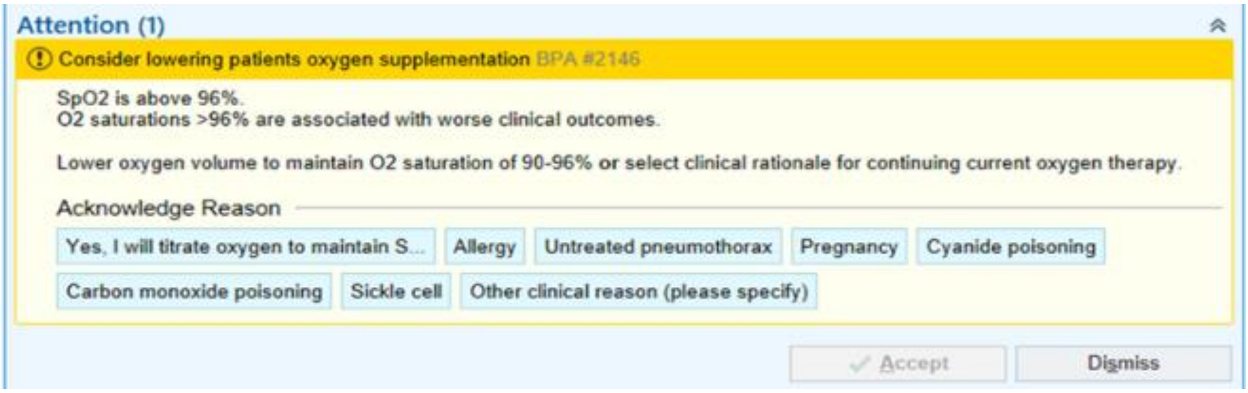

This figure displays the best practice alert (BPA) that would advise clinical care team members to down-titrate supplemental oxygen when their oxygen saturation was consistent with hyperoxemia (SpO2 > 96%). The BPA also provided options for possible clinical indications for maintaining hyperoxemia and current oxygen delivery.

eFigure 2. Multimodal Educational and Informatics Intervention Schematic

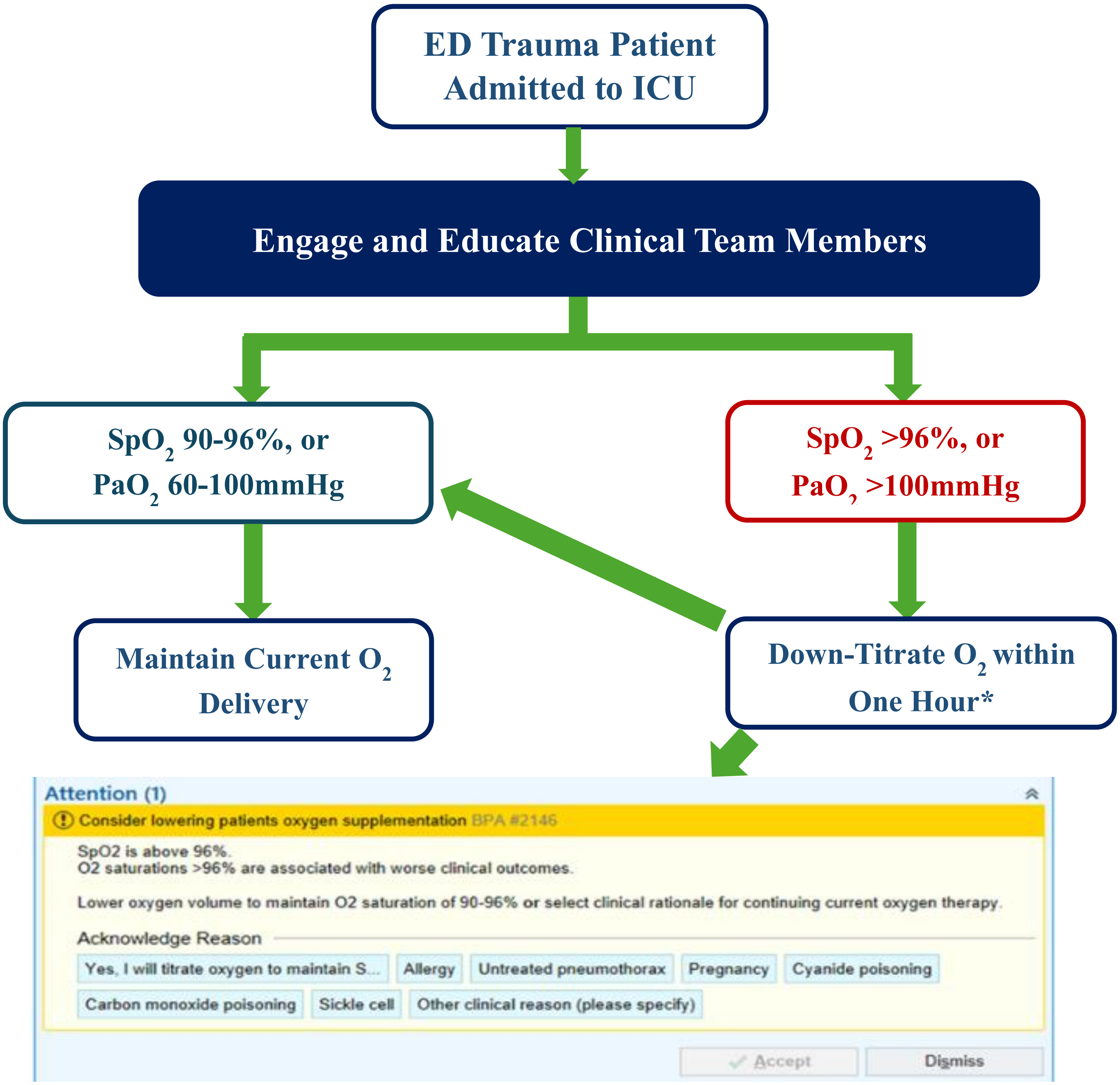

O2, oxygen; PaO2; partial pressure of arterial oxygen; SpO2, pulse oximeter oxygen saturation.

*Down-titration of oxygen was not clinically binding and could be overridden by the treating clinician. If, at any time, a treating clinician determined that an oxygenation target other than that assigned by the trial might be best for the patient's care, the oxygenation target for that patient could be modified.

© 2025 Douin DJ et al. *JAMA Network Open*

eFigure 3. Heterogeneity of Treatment Effect for Supplemental Oxygen Free Days (SOFD)

MD, mean difference; ICU, intensive care unit.

Subgroup	Usual Care	Intervention	Adjusted MD (95% CI)
Injury Severity Score			
>/= 16	15 (10.7)	17 (11)	0.39 (–0.38 to 1.16)
< 16	21 (8.67)	23.1 (7.96)	0.15 (–0.70 to 1.00)
Mechanical Ventilation			
Ventilated Before ICU Arrival	13.1 (10.7)	13.4 (11.2)	–0.40 (–1.25 to 0.44)
Not Ventilated Before ICU Arrival	20.6 (8.86)	22.6 (8.3)	0.75 (0.00 to 1.50)
Penetrating Mechanism of Injury			
Yes	17.7 (10)	18.8 (10.1)	0.06 (–1.09 to 1.22)
No	17.5 (10.4)	19.7 (10.3)	0.36 (–0.35 to 1.06)
Race and Ethnicity			
Non–Hispanic White	17.1 (10.5)	19.9 (10.2)	0.73 (–0.05 to 1.52)
Non–Hispanic Black	18.2 (9.72)	18.7 (9.88)	–0.01 (–1.20 to 1.17)
Hispanic	17.4 (10.1)	18.8 (10.1)	–0.18 (–1.58 to 1.22)
Other	16.6 (11)	18.5 (10.9)	0.45 (–1.07 to 1.98)
Shock Index >/= 1.0			
Yes	18.3 (10.1)	19.3 (10)	0.39 (–0.81 to 1.59)
No			0.36 (–0.34 to 1.06)
Traumatic Brain Injury	15.9 (11.6)	18.6 (11.2)	
Yes	18.3 (9.69)	20.4 (9.27)	0.59 (–0.26 to 1.45)
No			0.20 (–0.57 to 0.96)

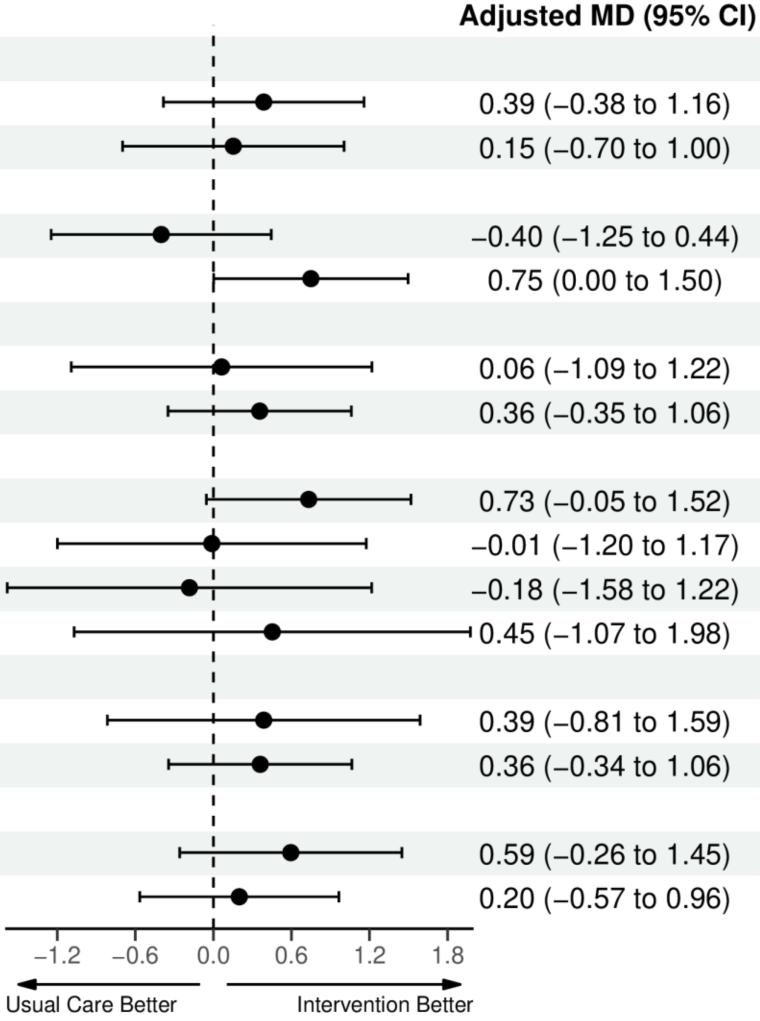

© 2025 Douin DJ et al. *JAMA Network Open*

eFigure 4. Sensitivity Analysis of Heterogeneity of Treatment Effect for Supplemental Oxygen-Free Days (SOFD) Excluding the One Site with Data Completeness Issues

Subgroup	Usual Care	Intervention	Adjusted MD (95% CI)
Injury Severity Score			
>/= 16	15.2 (10.9)	17.3 (11.1)	0.33 (–0.49 to 1.15)
< 16	21.1 (8.68)	23.3 (7.92)	0.10 (–0.61 to 0.81)
Mechanical Ventilation			
Ventilated Before ICU Arrival	13.1 (10.9)	13.4 (11.4)	–0.39 (–1.29 to 0.52)
Not Ventilated Before ICU Arrival	20.7 (8.86)	22.7 (8.31)	0.72 (0.10 to 1.34)
Penetrating Mechanism of Injury			
Yes	17.7 (10.2)	18.9 (10.4)	–0.04 (–1.29 to 1.21)
No	17.8 (10.5)	20.1 (10.3)	0.39 (–0.20 to 0.98)
Race and Ethnicity			
Non–Hispanic White	17.2 (10.6)	20.1 (10.2)	0.78 (–0.04 to 1.59)
Non–Hispanic Black	18.6 (9.65)	19.4 (9.8)	–0.02 (–1.05 to 1.01)
Hispanic	18.4 (10.1)	19.3 (10.2)	–0.50 (–2.08 to 1.08)
Other	17.3 (11.3)	19.6 (11.1)	0.89 (–0.85 to 2.63)
Shock Index >/= 1.0			
Yes	18.5 (10.2)	19.5 (10.1)	0.59 (–0.29 to 1.47)
No			0.18 (–0.46 to 0.83)
Traumatic Brain Injury	15.9 (11.6)	19 (11.2)	
Yes	18.6 (9.62)	20.8 (9.23)	0.88 (–0.46 to 2.21)
No			0.34 (–0.26 to 0.93)

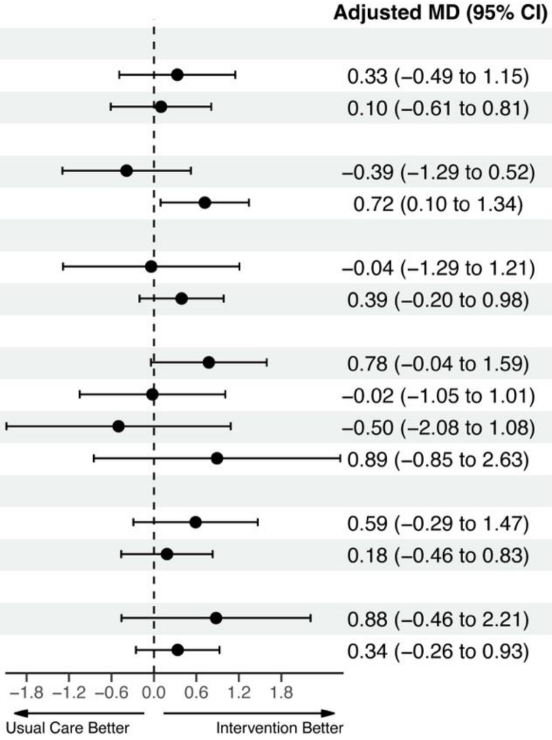

© 2025 Douin DJ et al. *JAMA Network Open*

eFigure 5. Sensitivity Analysis of Primary Outcome Model Specifications

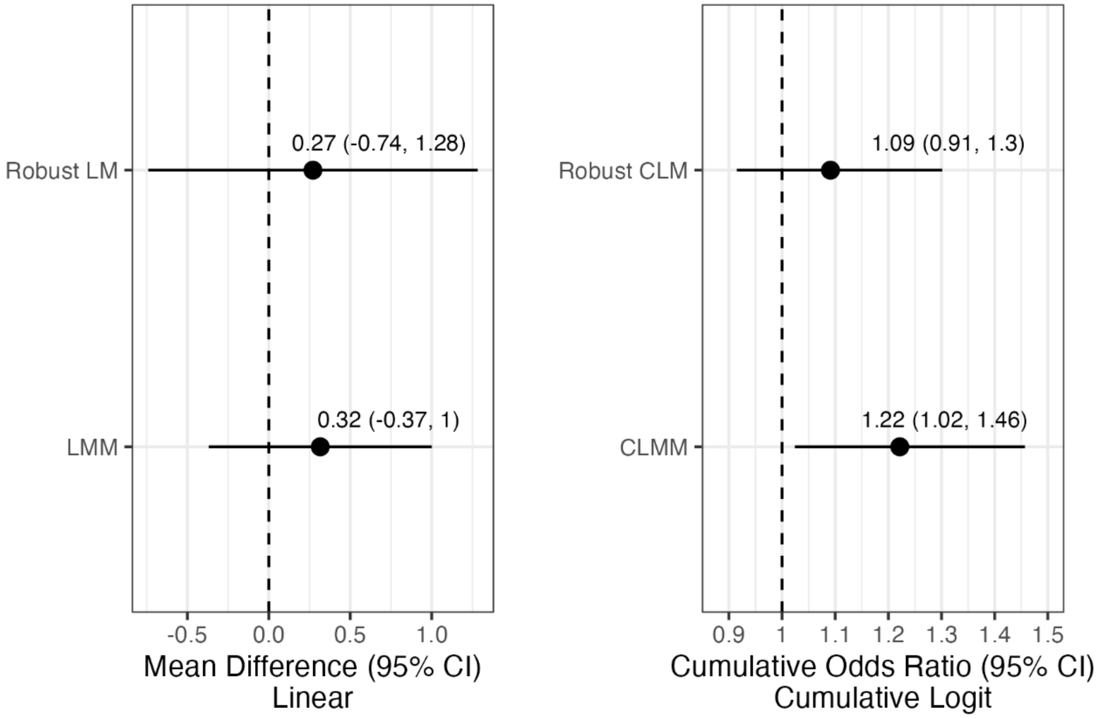

CI: confidence interval; CLM: cumulative logit model; CLMM: cumulative logit mixed model; LMM: linear mixed model (primary result).

© 2025 Douin DJ et al. *JAMA Network Open*

eFigure 6. SpO_2 and FiO_2 Stratified by Race/Ethnicity and Treatment Arm

FiO_2 and SpO_2 for Non-Hispanic Black Patients

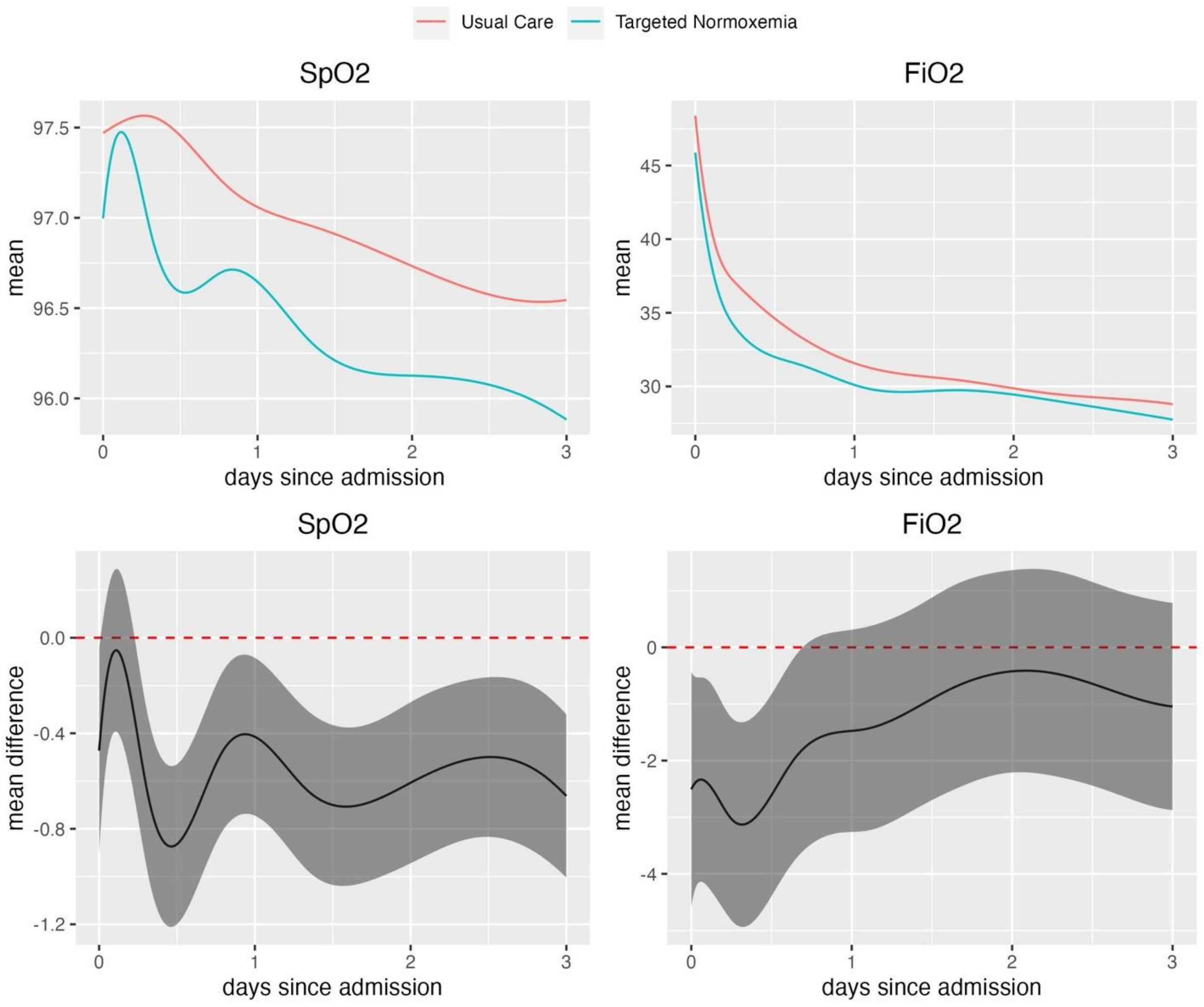

© 2025 Douin DJ et al. *JAMA Network Open*

FiO_2 and SpO_2 for Hispanic Patients

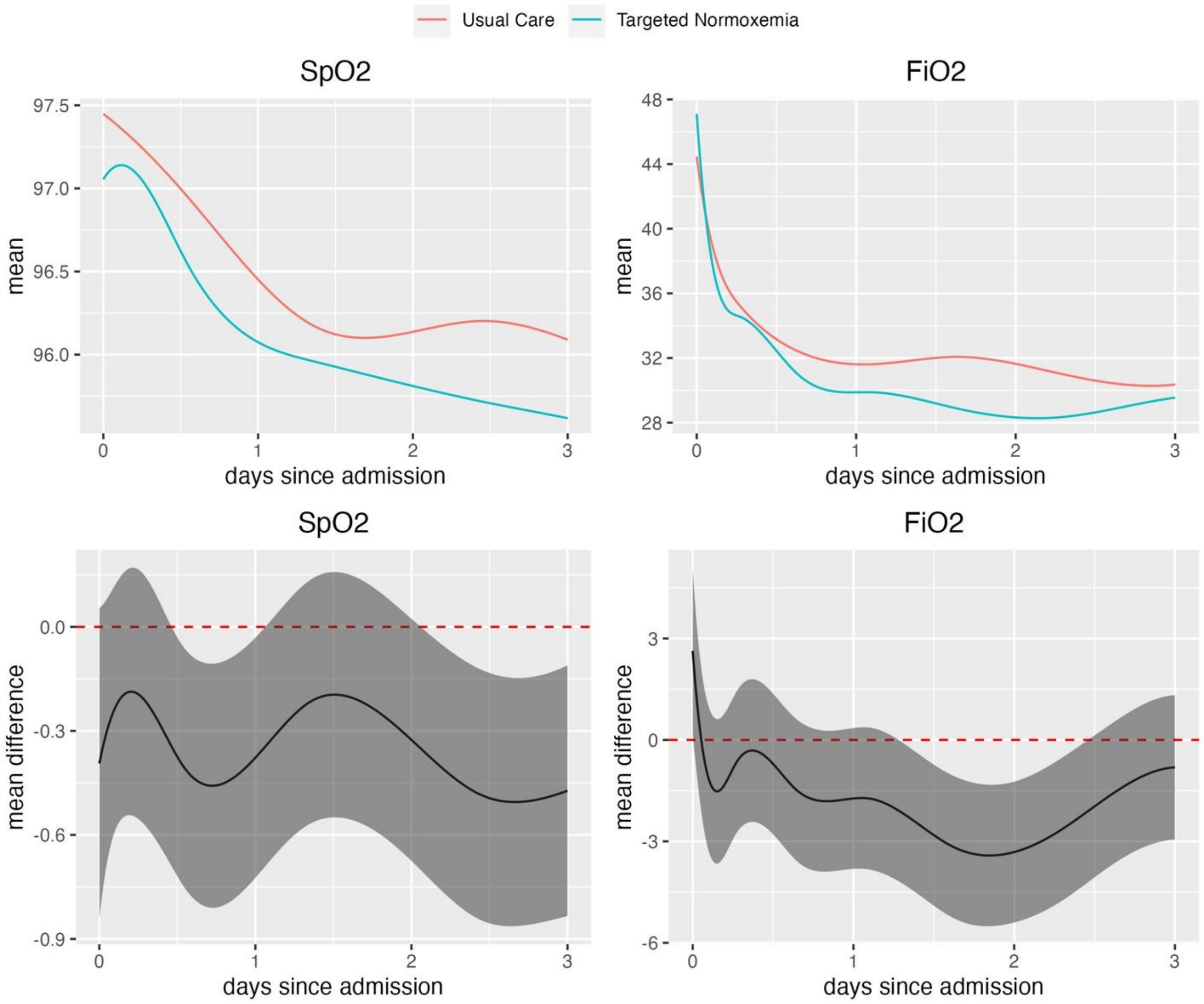

© 2025 Douin DJ et al. *JAMA Network Open*

FiO_2 and SpO_2 for Non-Hispanic White Patients

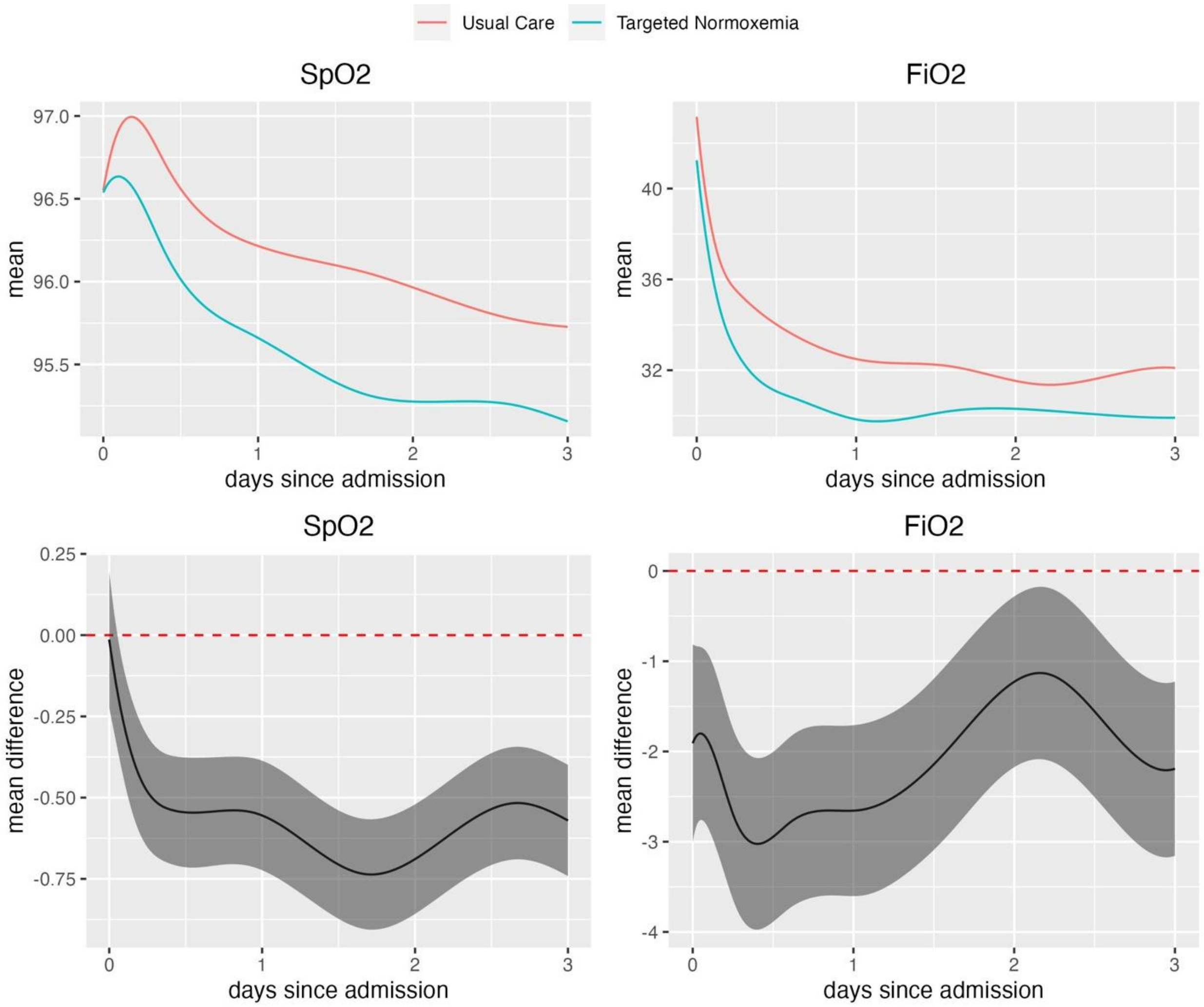

© 2025 Douin DJ et al. *JAMA Network Open*

FiO2 and SpO2 for Other Patients

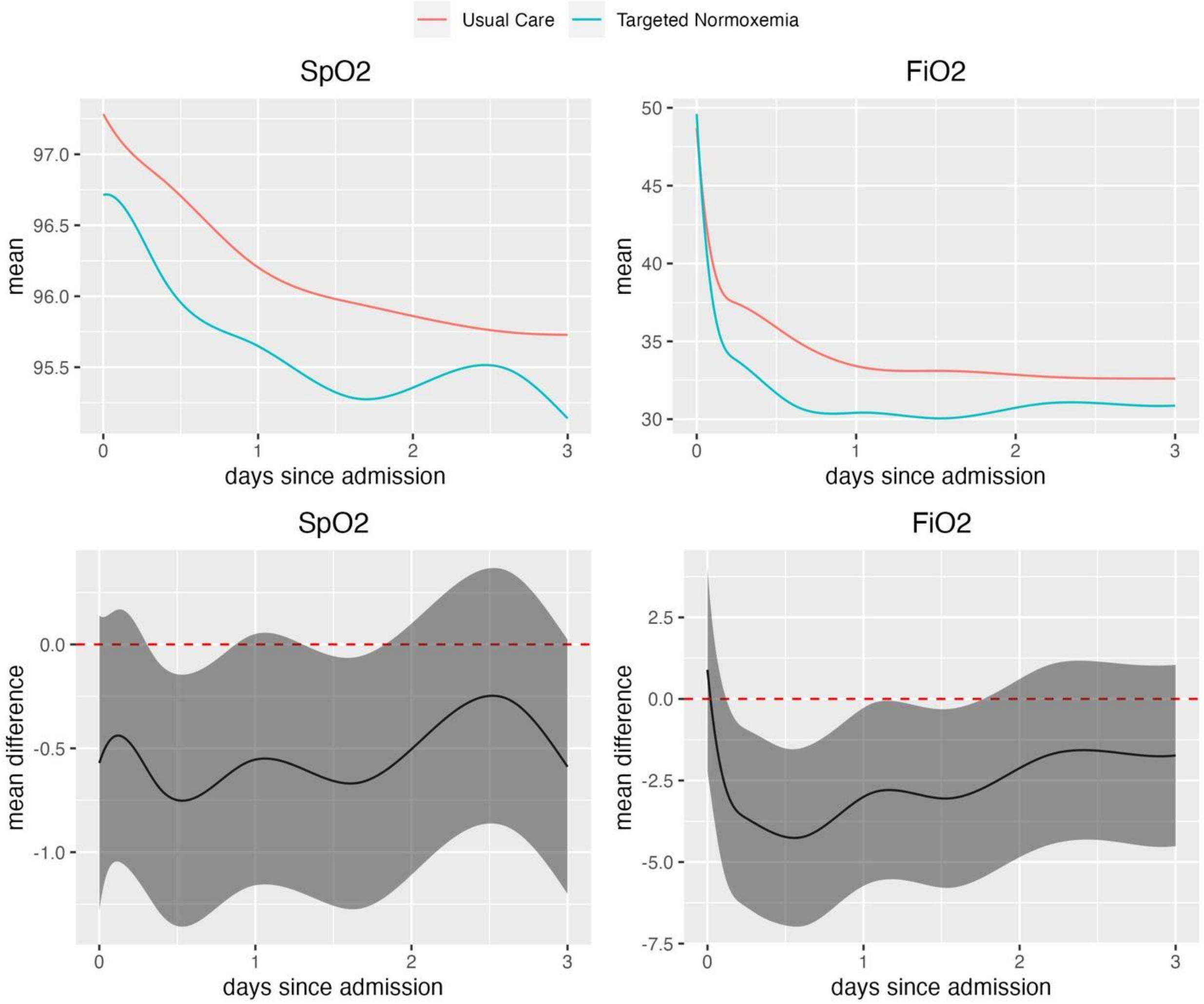

© 2025 Douin DJ et al. *JAMA Network Open*

eFigure 7. A) Density of patient time spent at fraction of inspired oxygen (FiO_2) and oxygen saturation (SpO_2) by group (targeted normoxemia vs. usual care) for modifiable patient time; B) Change in the density of patient time (targeted normoxemia minus usual care) spent at FiO_2 and SpO_2 for modifiable patient time

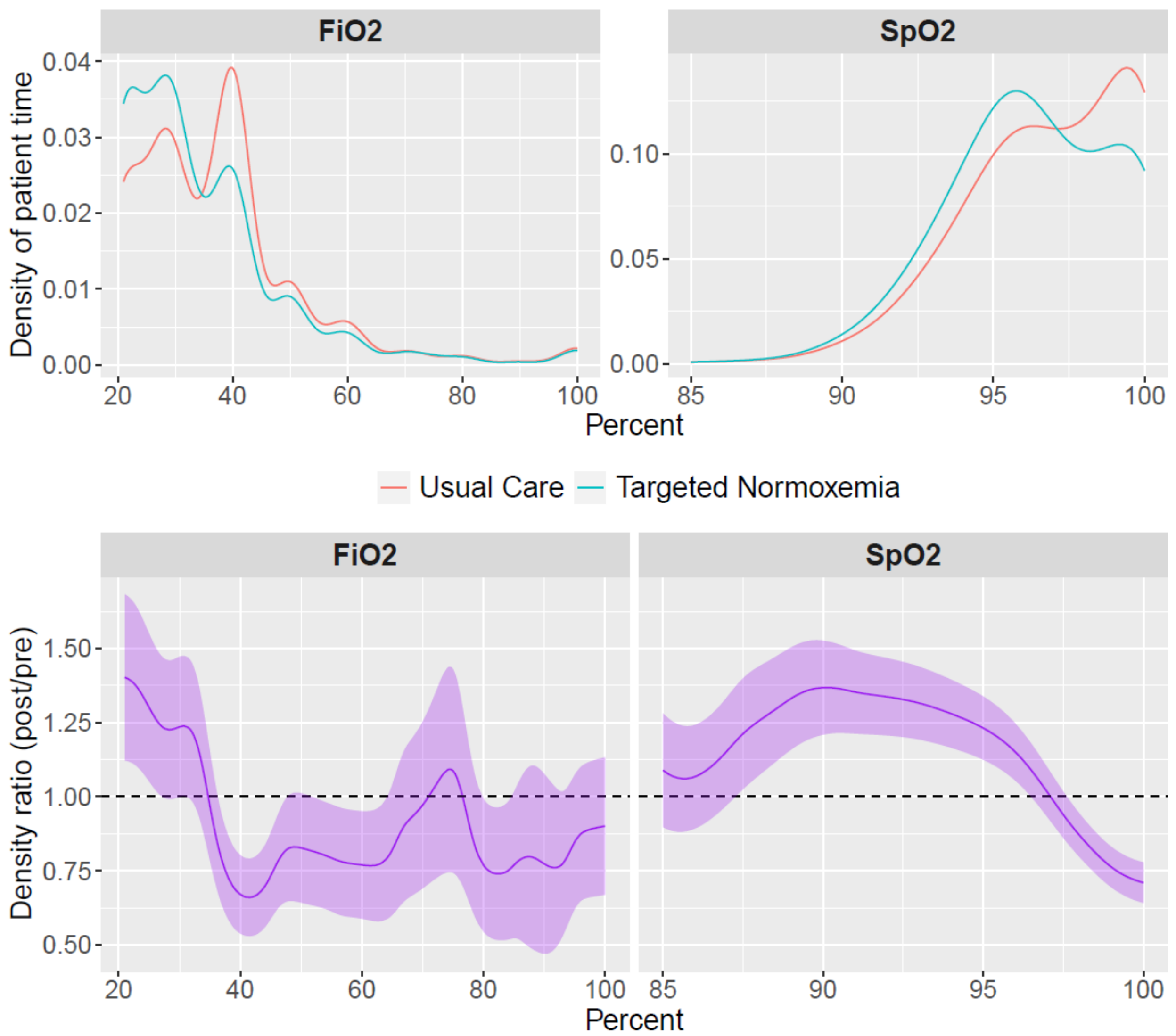

Shaded areas represent 95% confidence intervals. Areas in the bottom panels that have a ratio >1 indicate a shift from usual care to targeted normoxemia. For example, at 21% FiO2.

Patient time was considered “non-modifiable” if the patient was receiving room air or 21% FiO2 but had an oxygen saturation (SpO2) >96% (i.e., was in the hyperoxemia range). All other patient time is included in this figure.

© 2025 Douin DJ et al. *JAMA Network Open*

eFigure 8. A) Density of patient time spent at fraction of inspired oxygen (FiO_2) and oxygen saturation (SpO_2) by group (targeted normoxemia vs. usual care) for all patient time; B) Change in the density of patient time (targeted normoxemia minus usual care) spent at FiO_2 and SpO_2 for all patient time

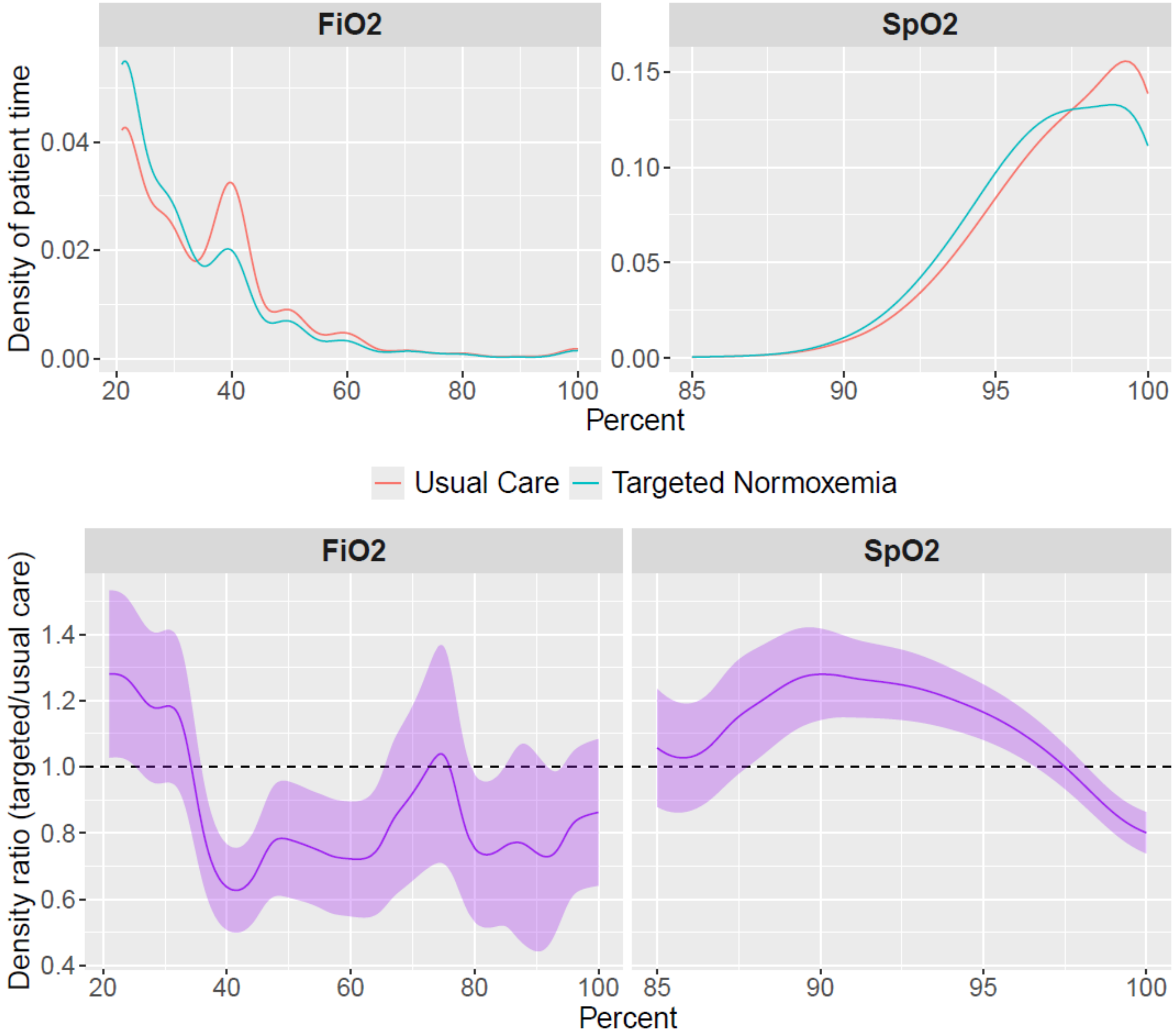

Shaded areas represent 95% confidence intervals. Areas in the bottom panels that have a ratio >1 indicate a shift from usual care to targeted normoxemia For example, at 21% FiO2.

© 2025 Douin DJ et al. *JAMA Network Open*

eFigure 9. Proportion of patients by quartile of supplemental oxygen-free days

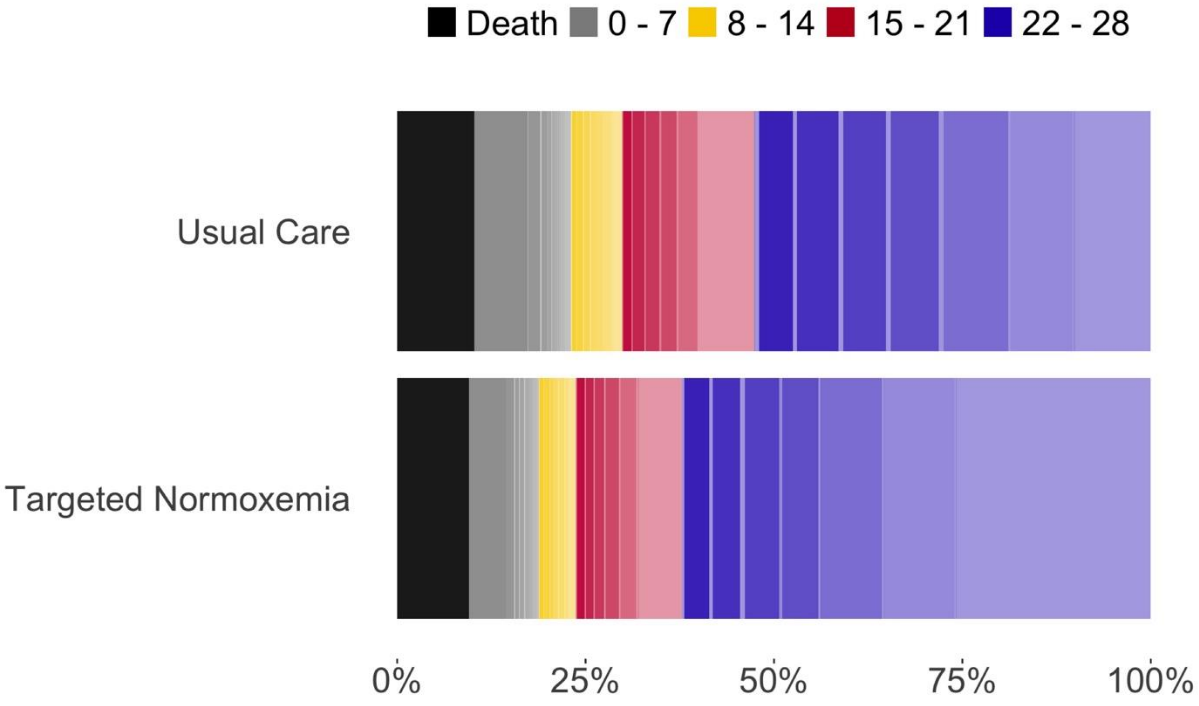

Each color represents the proportion of patients in one quartile of supplemental oxygen-free days through day 28. Each shade within each color represents the proportion of patients who achieved the exact number of supplemental oxygen-free days. Patients who experienced in-hospital mortality within 28 days of admission received a supplemental oxygen-free day value of -1 and are represented in black.

© 2025 Douin DJ et al. *JAMA Network Open*

eReferences

1. Douin DJ, Schauer SG, Anderson EL, et al. Systematic Review of Oxygenation and Clinical Outcomes to Inform Oxygen Targets in Critically Ill Trauma Patients. *Journal of Trauma and Acute Care Surgery*. May 30 2019;doi:10.1097/TA.0000000000002392
2. Schauer S, Anderson E, Cunningham C, Jones J, Bebarta V, Ginde A. Consensus-based recommendation for oxygenation targets in critically injured patients. Presented at Special Operations Medical Association Scientific Assembly; May 9, 2019. *Available at: http://wwwspecialoperationsmedicineorg/Documents/2019%20SOMSA/Handouts/THURS-Research%20Schauer%20-%20Oxygen%20consensuspdf*. 2021;
3. Dylla L, Anderson EL, Douin DJ, et al. A quasi-experimental study of targeted normoxia in critically ill trauma patients. *Journal of Trauma and Acute Care Surgery*. 2021;91(2S Suppl 2):S169-S175. doi:10.1097/ta.0000000000003177
4. Dylla L, Douin DJ, Anderson EL, et al. A multicenter cluster randomized, stepped wedge implementation trial for targeted normoxia in critically ill trauma patients: study protocol and statistical analysis plan for the Strategy to Avoid Excessive Oxygen (SAVE-O2) trial. *Trials*. Nov 8 2021;22(1):784. doi:10.1186/s13063-021-05688-6
5. Harris PA, Taylor R, Thielke R, Payne J, Gonzalez N, Conde JG. Research electronic data capture (REDCap)—A metadata-driven methodology and workflow process for providing translational research informatics support. *Journal of Biomedical Informatics*. 2009/04/01/ 2009;42(2):377-381. doi:https://doi.org/10.1016/j.jbi.2008.08.010
6. Cheng AC, Duda SN, Taylor R, et al. REDCap on FHIR: Clinical Data Interoperability Services. *Journal of Biomedical Informatics*. 2021/09/01/ 2021;121:103871. doi:https://doi.org/10.1016/j.jbi.2021.103871
7. Duprez F, Mashayekhi S, Cuvelier G, Legrand A, Reychler G. A New Formula for Predicting the Fraction of Delivered Oxygen During Low-Flow Oxygen Therapy. *Respir Care*. Dec 2018;63(12):1528-1534. doi:10.4187/respcare.06243
8. Dylla L, Douin DJ, Anderson EL, et al. A Multicenter Cluster Randomized, Stepped Wedge Implementation Trial for Targeted Normoxia in Critically Ill Trauma Patients: Study Protocol and Statistical Analysis Plan for Strategy to Avoid Excessive Oxygen (SAVE-O2) Trial. *Trials*. 2021;
9. Moore BJ, White S, Washington R, Coenen N, Elixhauser A. Identifying Increased Risk of Readmission and In-hospital Mortality Using Hospital Administrative Data: The AHRQ Elixhauser Comorbidity Index. *Med Care*. Jul 2017;55(7):698-705. doi:10.1097/MLR.0000000000000735
10. Little RJ, Rubin DB. *Statistical analysis with missing data*. vol 793. John Wiley & Sons; 2019.
11. RCoreTeam. R: A Language and Environment for Statistical Computing. R Foundation for Statistical Computing. https://www.R-project.org/.
12. Therneau T. A Package for Survival Analysis in R. R package version 3.2-3. https://CRAN.R-project.org/package=survival

© 2025 Douin DJ et al. *JAMA Network Open*
