# Supplementary material for: Targeted Normoxemia and Supplemental Oxygen–Free Days in Critically Injured Adults: A Stepped-Wedge Cluster Randomized Clinical Trial
Source: JAMA Netw Open. 2025 Mar 31;8(3):e252093. doi: 10.1001/jamanetworkopen.2025.2093 (PMC12186824; doi:10.1001/jamanetworkopen.2025.2093)
Supplement: Supplement 3. — Nonauthor Collaborators [file jamanetwopen-e252093-s003.pdf]

Supplemental Online Content: Nonauthor Collaborators
*First name, last name, and suffix (if applicable) are required and will appear in PubMed.

***Group Name(s): Strategy to Avoid Excessive Oxygen (SAVE-O2) Investigators**							
***First Name and Middle Initial(s)**	***Last Name**	***Suffix (eg, Jr, III)**	Academic Degrees	Institution	Location (city, state/province, country)	Role or Contribution, eg, chair, principal investigator	Group (if more than 1 Group listed in the byline) and/or Subgroup (eg, Steering Committee)
Luke	Hoffman		DO	United States Army Institute of Surgical Research	JBSA Ft. Sam Houston, Texas, USA	Co-Investigator	
Fabiola	Mancha		MS	United States Army Institute of Surgical Research	JBSA Ft. Sam Houston, Texas, USA	Research Coordinator	
Jessica	Mendez		MS	United States Army Institute of Surgical Research	JBSA Ft. Sam Houston, Texas, USA	Research Coordinator	
Stephanie	Gravitz		MPH	Denver Health Medical Center	Denver, Colorado, USA	Research Coordinator	
Emily	Hopkins		MSPH	Denver Health Medical Center	Denver, Colorado, USA	Research Coordinator	
Mia	Lundin		BS	Denver Health Medical Center	Denver, Colorado, USA	Research Coordinator	
Carolynn	Lyle		PA-C	Denver Health Medical Center	Denver, Colorado, USA	Co-Investigator	
Kelley	Mancine		MBA	Denver Health Medical Center	Denver, Colorado, USA	Research Coordinator	
Barry	Platnick		MD	Denver Health Medical Center	Denver, Colorado, USA	Co-Investigator	
Caitlin	Robinson		MPH	Denver Health Medical Center	Denver, Colorado, USA	Research Coordinator	
Ellie	Smith		MPH	Denver Health Medical Center	Denver, Colorado, USA	Research Coordinator	
Caitlyn	Hickey		RN	Oregon Health & Science University	Portland, Oregon, USA	Research Coordinator	
Laura	Nguyen		BS	Oregon Health & Science University	Portland, Oregon, USA	Research Coordinator	
Robel	Beyene		MD	Vanderbilt University Medical Center	Nashvile, Tennessee, USA	Co-Investigator	
Bradley	Lloyd		RRT-ACCS	Vanderbilt University Medical Center	Nashvile, Tennessee, USA	Co-Investigator	
Karen	Miller		RN, BSN	Vanderbilt University Medical Center	Nashvile, Tennessee, USA	Research Coordinator	
Mayur B.	Patel		MD, MPH	Vanderbilt University Medical Center	Nashvile, Tennessee, USA	Co-Investigator	
Shannon	Pugh		RN	Vanderbilt University Medical Center	Nashvile, Tennessee, USA	Research Coordinator	
Carolyn	Williams		RN	University of Alabama at Birmingham Medical Center and Heershink School of Medicine	Birmingham, Alabama, USA	Research Coordinator	
Michael	Kurz		MD	University of Alabama at Birmingham Medical Center and Heershink School of Medicine	Birmingham, Alabama, USA	Co-Investigator	
Alexa	DeRegnaucourt		MS	University of Cincinnati College of Medicine	Cincinnati, Ohio, USA	Research Coordinator	
Dina	Gomaa		MSc, RRT	University of Cincinnati College of Medicine	Cincinnati, Ohio, USA	Research Coordinator	
Julie A.	Phelps		BA	University of Cincinnati College of Medicine	Cincinnati, Ohio, USA	Research Coordinator	

Supplemental Online Content: Nonauthor Collaborators
*First name, last name, and suffix (if applicable) are required and will appear in PubMed.

***First Name and Middle Initial(s)**	***Last Name**	***Suffix (eg, Jr, III)**	Academic Degrees	Institution	Location (city, state/province, country)	Role or Contribution, eg, chair, principal investigator	Group (if more than 1 Group listed in the byline) and/or Subgroup (eg, Steering Committee)
John	Shinn		MSN, CNP	University of Cincinnati College of Medicine	Cincinnati, Ohio, USA	Research Coordinator	
Mitchell J.	Cohen		MD	University of Colorado School of Medicine	Aurora, Colorado, USA	Co-Investigator	
Jeffrey	Rixe		MD	University of Pittsburgh Medical Center	Pittsburgh, Pennsylvania, USA	Co-Investigator	
Jason	Sperry		MD, MPH	University of Pittsburgh Medical Center	Pittsburgh, Pennsylvania, USA	Co-Investigator	
Tina	Vita		RN, CCRC	University of Pittsburgh Medical Center	Pittsburgh, Pennsylvania, USA	Research Coordinator	
Nathan	Hoot		MD, PhD	University of Texas Health Science Center, Houston	Houston, Texas, USA	Co-Investigator	
Elizabeth	Vidales		MD, MPH, MBA	University of Texas Health Science Center, Houston	Houston, Texas, USA	Co-Investigator	
Charles E.	Wade		PhD	University of Texas Health Science Center, Houston	Houston, Texas, USA	Co-Investigator	
