# Supplementary material for: Targeted Normoxemia and Supplemental Oxygen–Free Days in Critically Injured Adults: A Stepped-Wedge Cluster Randomized Clinical Trial
Source: JAMA Netw Open. 2025 Mar 31;8(3):e252093. doi: 10.1001/jamanetworkopen.2025.2093 (PMC12186824; doi:10.1001/jamanetworkopen.2025.2093)
Supplement: Supplement 4. — Data Sharing Statement [file jamanetwopen-e252093-s004.pdf]

# Data Sharing Statement

Douin. Targeted Normoxemia and Supplemental Oxygen–Free Days in Critically Injured Adults. *JAMA Netw Open*. Published March 31, 2025. doi:10.1001/jamanetworkopen.2025.2093

**Data**
**Additional Information:** NCT04534959
**Data available:** No

**Additional Information**
**Explanation for why data not available:** Per Department of Defense regulations and associated data sharing agreements with sites, we cannot share data outside the University of Colorado coordinating center. However, we will certainly be willing to collaborate with outside investigators on secondary analyses, upon reasonable request.
